# Lewis-acid activation of persulfate enables oxidation of antibiotics: a new green low-cost advanced oxidation process

**DOI:** 10.1039/d6ra06842a

**Published:** 2026-10-21

**Authors:** Anushka Lucas, Haya Kornweitz, Yael Albo, Dan Meyerstein

**Affiliations:** a Chemical Sciences Department, The Radical Research Center, Ariel University Ariel Israel hayak@ariel.ac.il danm@ariel.ac.il; b Chemical Engineering Department, The Radical Research Center, Ariel University Ariel Israel yaelyt@ariel.ac.il; c Chemistry Department, Ben-Gurion University Beer-Sheva Israel

## Abstract

Developing advanced oxidation processes (AOPs) that operate efficiently under environmentally relevant conditions remains a key challenge in water treatment. The results presented herein demonstrate that the –O–O– bond in persulfate is activated to react with substrates by forming (H_2_O)_4_Mg(OS(O)_2_OOSO_3_)/H(OS(O)_2_OOSO_3_)^−^ that oxidise antibiotics (ciprofloxacin and ofloxacin). At pH 7.4, Mg^2+^/PDS achieved 75% degradation of ciprofloxacin and 89% degradation of ofloxacin after 24 h, with pseudo-first-order rate constants of 2.7 × 10^−3^ and 5.0 × 10^−4^ min^−1^, respectively. These values represent over a twentyfold enhancement over PDS alone at the same pH. In addition, for the reaction with DMSO, a novel mechanism was found, in which the oxidation processes are initiated by Lewis acid activation and do not involve initial radical formation; the substrates react directly with the activated O–O bond. Thus, its reactions with sulfoxides form the corresponding sulfones. This mechanism, for the reaction with DMSO is supported by DFT calculations. In contrast, the calculations indicate a radical mechanism for the reaction with phenol, suggesting that the substrate oxidation mechanism depends on the nature of the substrate. The results prove that peroxides can be activated towards oxidation processes not only *via* energy transfer (*e.g.*, heat or light) or redox processes (*e.g.*, Fenton-like or electrochemistry) but also *via* Lewis acid activation. The results establish an additional pathway for peroxide activation and provide a mechanistic basis for further exploring the development of potentially cost-effective and environmentally compatible advanced oxidation processes. This study highlights magnesium and protons as effective activators of persulfate for robust contaminant degradation in aqueous systems.

## Abstract 2

A new, low-cost oxidation process uses environmentally benign magnesium complexes to activate persulfate without radical intermediates. This drives robust degradation of fluoroquinolone antibiotics *via* selective oxygen atom transfer.

## Graphical abstract

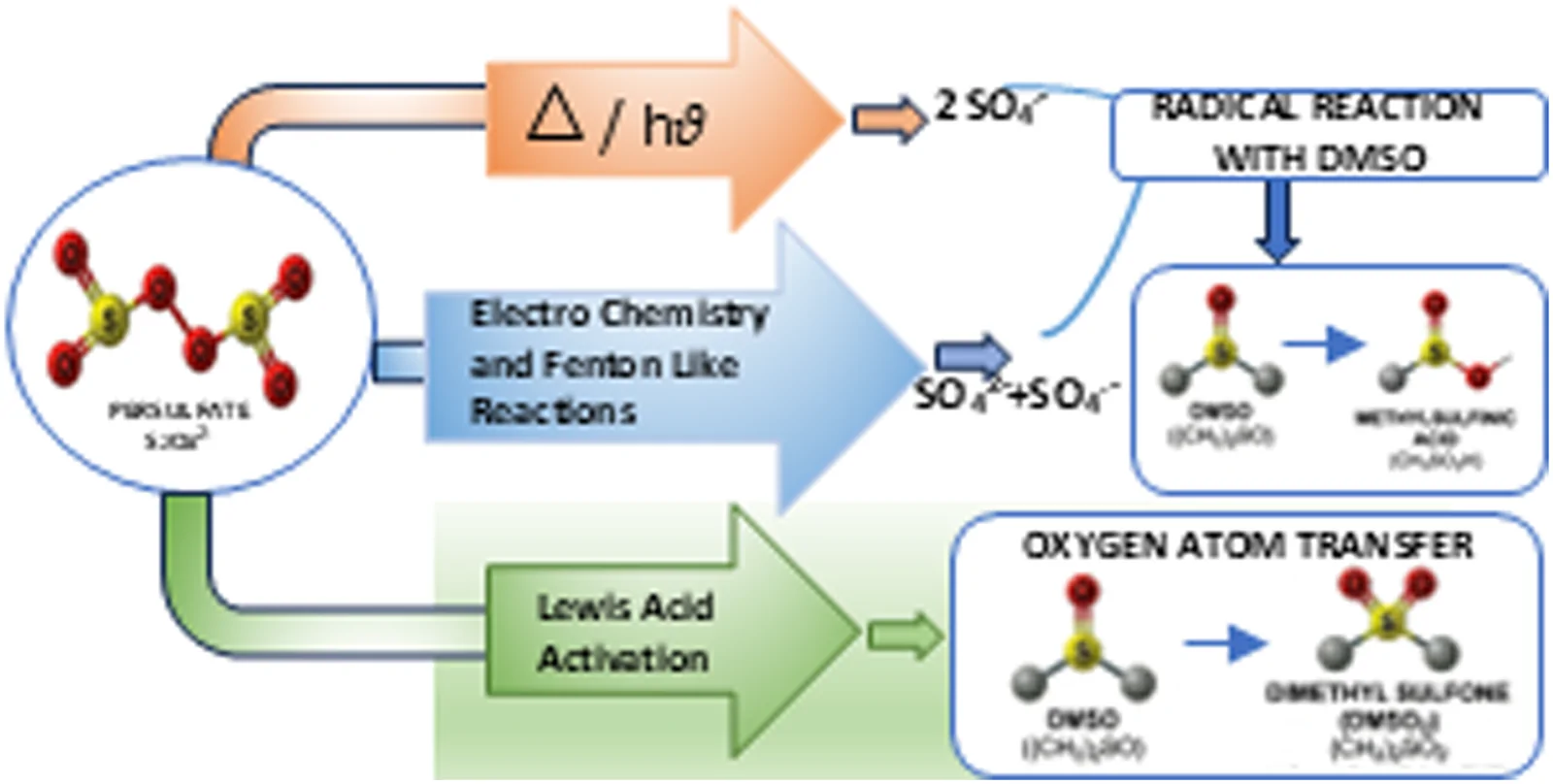

## 1 Introduction

Advanced oxidation processes (AOPs) are among the most efficient options for removing organic pollutants,^1,2^ including pharmaceutically active compounds from aqueous systems.^3,4^ Among different types of AOPs, Fenton's reaction is the widely used method because of its simplicity and it being environment-friendly and cost-effective.^5^ Fenton and Fenton-like reactions are primarily performed using Fe^2+^ or other low-valent transition-metal complexes.^5^ Using transition metal complexes comes with challenges such as precipitation of metal oxides and metal hydroxides, *e.g.*, Fe(OH)_3_, that can lead to sludge formation and hinder the efficiency of the process.^6^ There have been many studies that are aimed at finding a replacement for these transition metal complexes with other metals such as cobalt, manganese, beryllium, and aluminium, but these systems have a disadvantage: they work only in organic solvents and very small quantities of water and require a very high concentration of hydrogen peroxide to function.^7–10^

Pharmaceutical wastes that are active upon discharge into water pose a major threat to aquatic life and the water environment.^11,12^ The consumption of antibiotics is increasing due to population growth, development in medical science, and new-age diseases. Antibiotic consumption worldwide over the last decade has increased by approximately 20–30%.^13,14^ Antibiotic use in livestock, particularly cattle, poultry, and swine, has remained high, with a global yearly increase of roughly 5–10%.^15,16^ Nearly one-third of all antibiotics used globally are detected in wastewater and river systems.^17^ These materials enter the cycle either *via* pharmaceutical industry wastewater or through urban wastewater that basically contains faeces, urine, and incorrect disposal of unused or outdated medicines.^18–20^ This results in the development of antimicrobial resistance among bacteria, which further slows down the effectiveness of these antibiotics and the research in the development of new drugs.^21^ Moreover, the transformation of drugs in the environment may sometimes generate harmful byproducts.^22,23^ As the population is growing, the need to reuse wastewater for industrial and agricultural activities, and in some cases to obtain water for drinking, highlights the need to develop advanced treatment methods that can safely remove these compounds.^24^

Ciprofloxacin (CIP) and ofloxacin (OFX) are fluoroquinolone (FQ) antibiotics that are prescribed to treat bacterial infections such as Urinary Tract Infections (UTIs),^25^ chest infections (including pneumonia), and skin and bone infections.^26^ CIP is also used to help prevent developing meningitis.^27^ OFX is also used to treat chlamydia.^28^ These two drugs, individually or in combination with other drugs, constitute various pharmaceutical formulations.^29^ The removal of CIP and OFX is important, as prolonged exposure to these antibiotics can cause health problems both in humans and animals,^30^ like liver damage^31^ and central nervous system abnormalities.^32^ A recent study shows that 75% of used CIP remains unmetabolized and is excreted, leading to pollution of surface water, groundwater, and even drinking water.^33,34^ So, direct degradation of these pollutants in the water phase is the most desirable approach. Conventional oxidation technologies, such as UV radiation, chlorination, and ozonation, are among the most widely used treatment methods,^35^ but they are not always successful in degrading persistent antibiotic contaminants.^35,36^ To overcome these limitations, advanced oxidation Processes (AOPs) have emerged as promising alternatives.

Recent evidence indicates that Mg^2+^, despite being redox-inactive, can function as a transition-metal-free catalyst to activate peroxides under mild conditions.^37,38^ In the presence of hydrogen peroxide, peroxy-mono-sulfate (PMS) and persulfate (PDS), magnesium can act as a Lewis acid, facilitating reactions that generate reactive oxygen species.^39–41^

Fluoroquinolone antibiotics, such as CIP and OFX, are persistent pharmaceutical contaminants frequently detected in wastewater and natural aquatic environments due to their structural stability and resistance to conventional biological treatment.^40–42^ Their molecular structures feature an electron-rich aromatic quinolone core and a piperazine ring, which are particularly susceptible to oxidation by selective radicals such as CO_3_˙^−^ and SO_4_˙^−^.^40,42^ Moreover, at circumneutral pH, these antibiotics primarily exist as zwitterions or cations,^43,44^ which minimizes competitive binding to Mg^2+^, ensuring that the cation remains available as a coordination template for efficient peroxide activation.^45,46^

Motivated by these mechanistic and environmental considerations, the present study systematically investigates the Mg^2+^/PDS system to evaluate its capacity to degrade CIP and OFX. By monitoring the time-dependent decrease in substrate concentration, the efficiency and selectivity of magnesium-mediated oxidation are assessed under environmentally relevant conditions. In addition, this study examines the pH-dependent behaviour of peroxy-disulfate, providing insight into its reactivity across different pH regimes and identifying optimal conditions for achieving maximum degradation efficiency.

## 2 Methods

### 2.1 Experimental procedures and analytical methods

Experimental procedures were conducted in 100 mL glass beakers under constant magnetic stirring to evaluate the degradation of CIP and OFX (0.25 mM) using magnesium (Mg) and potassium peroxy-disulfate (PDS). Unless otherwise specified, the degradation experiments were conducted under aerated conditions without prior de-aeration or inert-gas purging. The reaction kinetics were monitored *via* UV-Vis spectrophotometry, while pH levels were precisely maintained using 1 M HClO_4_ and 0.1 M NaOH. Quantitative analysis of antibiotic degradation was performed using a Dionex Ultimate 3000 HPLC system (Thermo Scientific) equipped with a Diode Array Detector and a Hypersil GOLD column (150 × 4.6, particle size-3 µm). The mobile phase consisted of 77% 0.02 M phosphate buffer and 23% acetonitrile (ACN) at a flow rate of 1.50 mL min^−1^, with the column temperature maintained at 25 °C and detection set at 277 nm. The chromatographs of the calibration standards are presented in Fig. S1. To investigate the Oxygen Atom Transfer (OAT) mechanism, dimethyl sulfoxide (DMSO) was employed as a chemical probe, with the resulting transformation to dimethyl sulfone (DMSO_2_) confirmed through ^1^H spectra (400 MHz), which were recorded on a Bruker Advance III HD NMR Spectrometer equipped with a 5 mm BBFO probe and *Z*-axis gradient coil. The samples were dissolved in a mixture of 90% H_2_O/10% D_2_O for NMR spectroscopy.

All kinetic experiments were performed in triplicate, and the results are reported as mean ± standard deviation (SD). Pseudo-first-order rate constants (*k*_obs_) were determined from the kinetic data by regression analysis. Error bars in the quantitative figures represent ±SD.

### 2.2 Computational details

Gaussian16 (ref. 47) Software was used to perform DFT calculations, using Becke Three-Parameter Hybrid Functional B3LYP^48^ with the all-electron basis-set 6-311 + G(d,p).^49^ In all calculations, Grimme's D3BJ dispersion with Becke–Johnson damping^50^ was used. Truhlar's variation of the IEFPCM implicit solvation model *via* the SMD option^50^ was used to include solvation effects. Free energy values, including zero-point energies (ZPE) and thermal corrections, in standard conditions (*T* = 298 K, *P* = 1 atm, concentration = 1 M) were calculated. A vibrational frequency analysis was performed for each optimized structure to calculate the ZPE, verify that each stationary point is a real minimum (no imaginary frequencies), and identify transition states (TS) (single imaginary frequency). To locate the transition states, a relaxed potential energy surface scan followed by optimization to a transition state using the OPT = TS (the Berny algorithm) or QST3 (ref. 51 and 52) keyword was performed.

The 3D figures were captured using Chemcraft software.^53^

## 3 Results and discussion

### 3.1 Experimental results

The mechanistic foundation of the present study is based on the MgCl_2_·6H_2_O + HCO_3_^−^ + H_2_O_2_ system, which exhibits efficient degradation of methylene blue under circumneutral conditions. The reaction follows pseudo-first-order kinetics with respect to both H_2_O_2_ and HCO_3_^−^. In that study, the active species was assigned to the (H_2_O)_5_Mg-OC(O)OOH^+^ complex.^38^ It seemed of interest to check whether Mg(H_2_O)_6_^2+^ also activates oxidations by PDS and if so, *via* which mechanism.

The degradation of CIP and OFX was used as a model system to probe reactivity in the system MgCl_2_·6H_2_O + PDS. The results presented in Fig. 1 clearly indicate that indeed Mg^2+^ catalyses the degradation of both antibiotics by PDS. The corresponding HPLC chromatograms are provided in Fig. S2–S5.

**Fig. 1:**
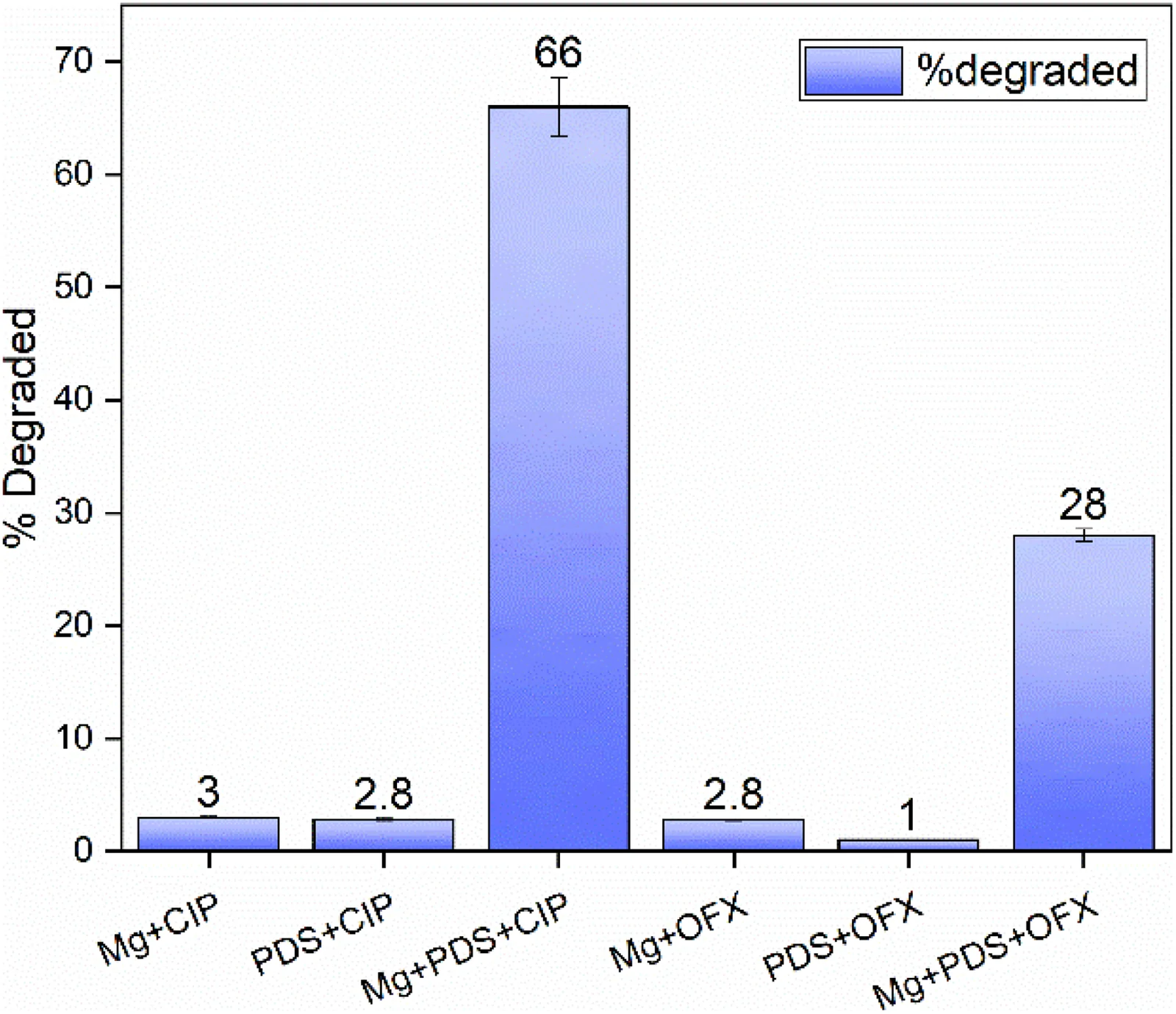
Removal efficiency of (CIP) and (OFX) for the systems: Mg^2+^ + CIP, PDS + CIP, Mg^2+^ + PDS + CIP and Mg^2+^ + OFX, PDS + OFX, Mg^2+^ + PDS + OFX. ([PDS] = 20 mM, [MgCl_2_·6H_2_O] = 2.0 mM, [CIP], [OFX] = 0.25 mM). All samples were at pH 7.40, aerated, and analysed by HPLC after 24 h of preparation.

As chloride might be involved in the oxidation processes *via* formation of reactive intermediates, *e.g.*, Cl˙, Cl_2_˙^−^ HClO/ClO^−^, the study was reproduced using MgSO_4_. The results presented in Fig. 2(a) indicate that the rate of CIP degradation is only slightly slower in the absence of chloride. Surprisingly, the degradation of OFX is slightly faster in the sulfate-containing system, Fig. 2(b). As CIP is a zwitterion at pH 7.4 (ref. 54 and 55) and OFX is still partially positively charged^56^ it was decided to repeat the OFX study at pH 8.5, where it is a zwitterion. The results presented in Fig. 2(c) clearly demonstrate that the observed differences between Cl^−^ and SO_4_^2−^ in this system are due to variation in ionic strength rather than to the oxidation of chloride ions. The results presented in Fig. 2(a) and (c) are attributed to the effect of ionic strength on the equilibrium.

**Fig. 2:**
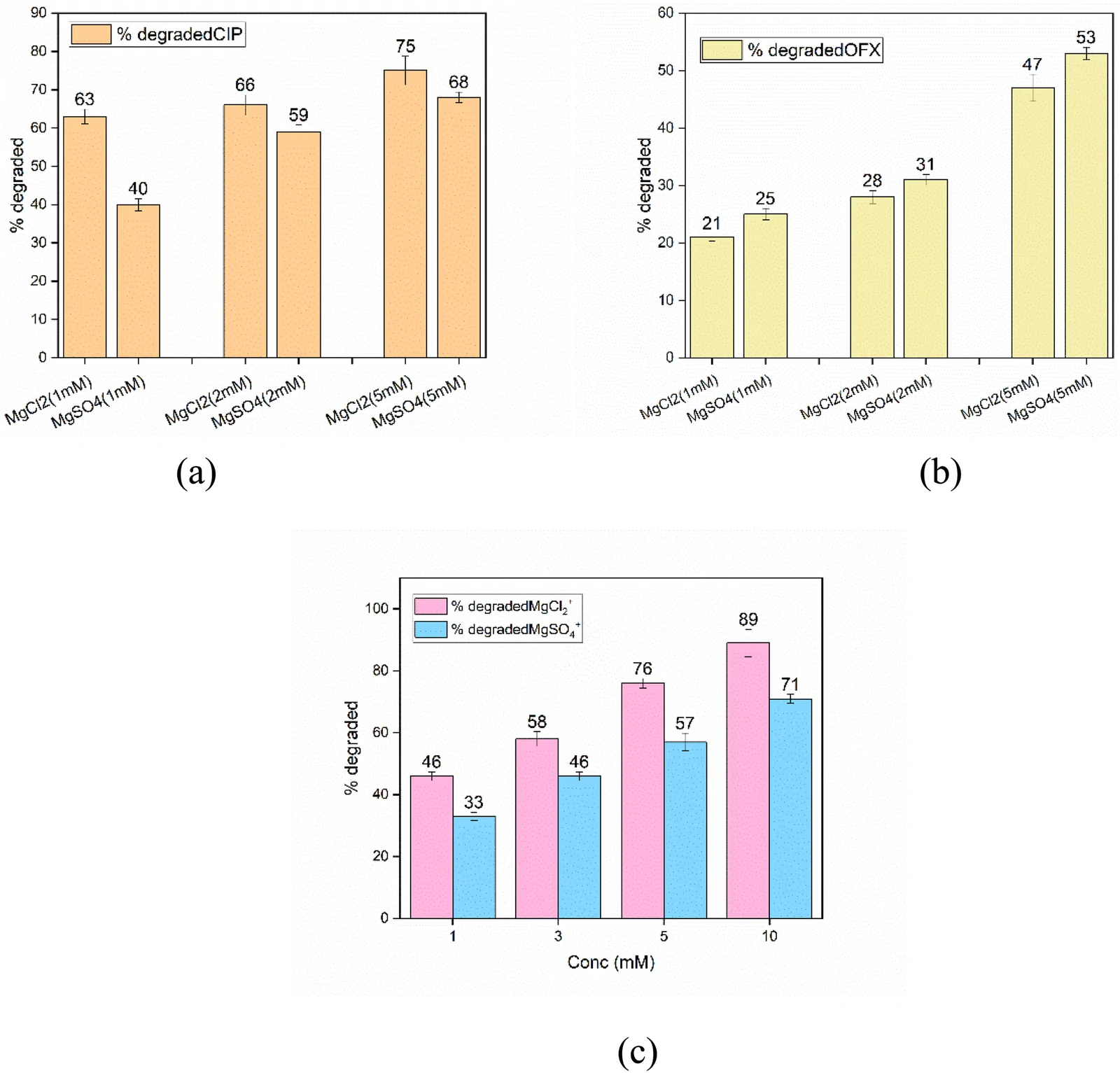
Comparative removal efficiencies by MgCl_2_ and MgSO_4_ in aerated solutions by [PDS] = 20 mM of (a) [CIP] = 0.25 mM, pH 7.4 (b) [OFX] = 0.25 mM, pH 7.4 (c) [OFX] = 0.25 mM, pH 8.5 All samples are aerated and analysed by HPLC after 24 h of preparation.

Clearly, an increase in the ionic strength decreases the concentration of (H_2_O)_4_Mg(OS(O)_2_OOS(O)_3_) that initiates the processes studied (for the structure of (H_2_O)_4_Mg(OS(O)_2_OOS(O)_3_) see DFT derivation below). The results presented in Fig. 2(b) are tentatively attributed to the suggestion that the transition state of the degradation step requires that the positive charged part of OFX has to be near the Mg^2+^ in the complex. This is clearly facilitated by an increase in the ionic strength.

Once Mg^2+^ was confirmed to act as an activator of persulfate, it was decided to check whether the degradation rates are pH-dependent. It should be noted that the kinetic experiments used to determine the pseudo-first-order rate constants in Table 1 were monitored over approximately 1 h, whereas Fig. 1 represents endpoint degradation measured after 24 h. The shorter monitoring period was selected for the kinetic experiments because the reactions, particularly under acidic conditions, proceed rapidly. Therefore, the kinetic data in Table 1 and the 24 h removal efficiencies in Fig. 1 represent different experimental time scales and should not be directly compared. The kinetic results are shown in Fig. S6 and summarized in Table 1. As the results indicate that the observed rate constants increase considerably with the decrease in pH, it was decided to determine whether degradation also occurs in the absence of Mg^2+^. Table 1 presents the pseudo-first-order rate constants for the degradation of CIP and OFX across a pH range of 3.25 to 7.4. It specifically compares the efficiency of peroxy-disulfate (PDS) alone with that of the magnesium-activated peroxy-disulfate system (Mg^2+^ + PDS). The results prove that indeed HS_2_O_8_^−^ reacts *via* an analogous mechanism to that of (H_2_O)_4_Mg-OS(O)_2_OOS(O)_3_. Across the entire pH range investigated, the observed rate constant in solutions containing Mg^2+^ is larger than in its absence. But the ratio between the two values changes dramatically from *ca.* 1.2 at pH 3.5 to *ca.* 5 at pH 7.4.

**Table 1:** Pseudo-first-order rate constants (*k*_obs_) for the degradation of CIP and OFX by PDS and Mg^2+^/PDS at different pH values. Rate constants were determined from kinetic measurements monitored for approximately 1 h. Values are reported as mean ± SD from independent experiments

pH	CIP + (PDS) *k*_obs_ (× 10^−2^ min^−1^), mean ± SD	OFX + (PDS) *k*_obs_ (× 10^−2^ min^−1^), mean ± SD	CIP (Mg^2+^ + PDS) *k*_obs_ (× 10^−2^ min^−1^), mean ± SD	OFX (Mg^2+^ + PDS) *k*_obs_ (× 10^−2^ min^−1^), mean ± SD
3.25	0.590 ± 0.016	0.170 ± 0.006	1.990 ± 0.064	0.290 ± 0.012
3.50	2.900 ± 0.111	0.580 ± 0.018	3.600 ± 0.100	0.660 ± 0.024
3.80	5.100 ± 0.164	0.700 ± 0.029	6.200 ± 0.238	1.660 ± 0.051
4.00	4.300 ± 0.119	0.950 ± 0.034	8.800 ± 0.283	1.940 ± 0.081
4.20	3.500 ± 0.135	0.630 ± 0.019	7.000 ± 0.194	1.470 ± 0.053
4.40	2.400 ± 0.077	0.330 ± 0.014	5.000 ± 0.192	0.920 ± 0.028
4.60	1.400 ± 0.039	0.230 ± 0.008	3.530 ± 0.113	0.740 ± 0.031
4.80	0.880 ± 0.034	0.1800 ± 0.055	2.500 ± 0.069	0.650 ± 0.023
5.00	0.770 ± 0.025	0.140 ± 0.006	2.100 ± 0.081	0.520 ± 0.016
6.00	0.530 ± 0.015	0.080 ± 0.003	1.330 ± 0.043	0.310 ± 0.013
7.00	0.180 ± 0.007	0.030 ± 0.001	0.750 ± 0.021	0.180 ± 0.006
7.40	0.050 ± 0.002	0.010 ± 0.0003	0.270 ± 0.010	0.050 ± 0.002

It should be noted that the kinetic experiments used to determine the pseudo-first-order rate constants (*k*_obs_) presented in Table 1 were monitored for approximately 1 h, whereas Fig. 1 represents endpoint degradation measured after 24 h. The *k*_obs_ values therefore describe the initial kinetic regime and were not extrapolated to predict the 24 h removal efficiencies. All kinetic experiments were performed independently, and the rate constants are reported as mean ± standard deviation (SD). Consequently, the kinetic data in Table 1 and the 24 h removal efficiencies in Fig. 1 represent complementary measurements obtained over different experimental timescales and should not be directly compared by extrapolation.

The results clearly point out that both Mg(H_2_O)_6_^2+^ and H_3_O^+^ catalyse the degradation of both antibiotics. The contribution of each catalyst also depends on pH, as the nature of the antibiotics is pH-dependent: p*K*_a1_ = 6.1 for CIP^57^ and p*K*_a1_ = 6.4 for OFX.^58^ The slight increase in the observed rate constants when the pH is raised from 3.25 to 4.0 is attributed to the start of the formation of the zwitterionic forms of the antibiotics. At higher pH values, the loss of H_3_O^+^ dominates. As the product of the reaction with DMSO at pH 4.0 is also DMSO_2_ (Fig. 3), the results indicate that HS_2_O_8_^−^ is the catalyst and that the processes observed are initiated by:

1 HS_2_O_8_^−^/(H_2_O)_4_Mg-OS(O)_2_OOS(O)_3_ + substrate → HSO_4_^−^/{(H_2_O)_5_Mg-OS(O)_3_^−^ + H^+^} + substrate-OS(O)_3_

**Fig. 3:**
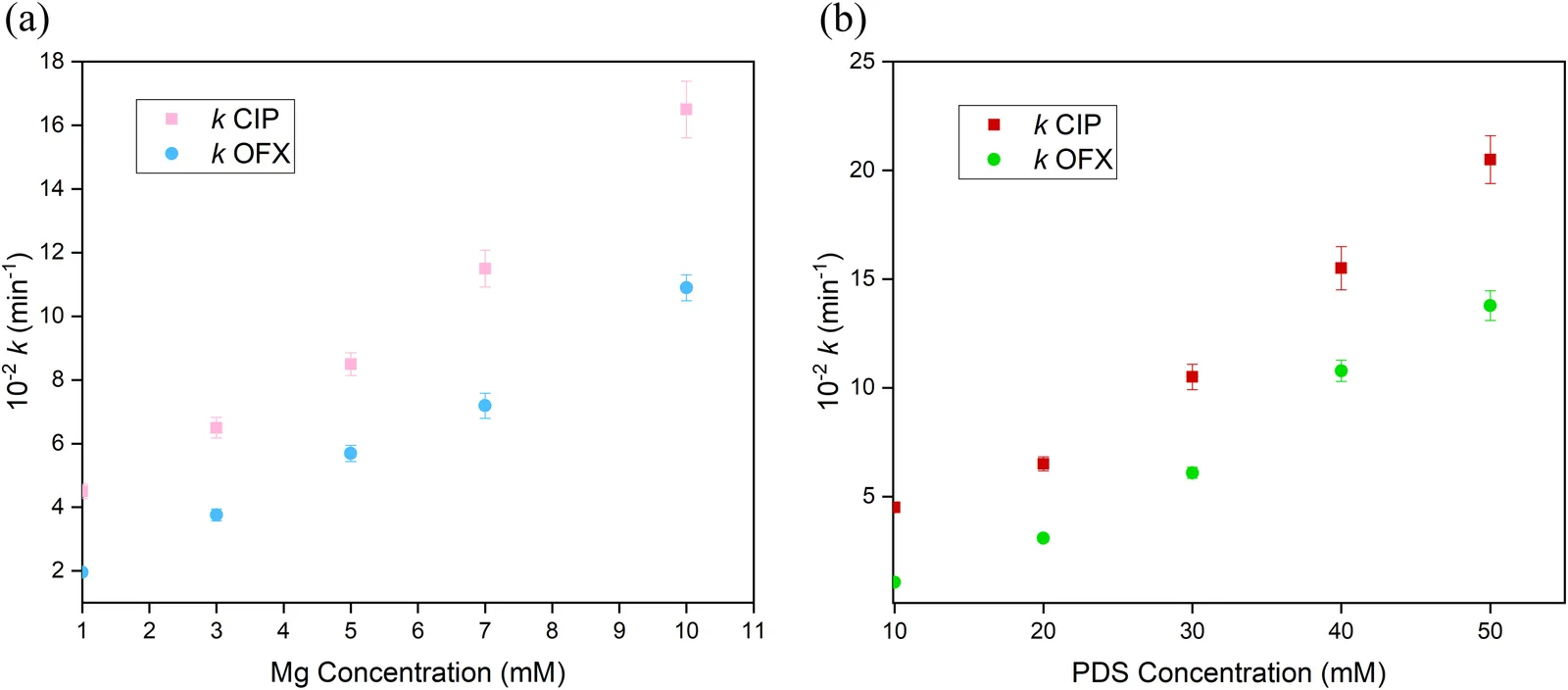
Dependence of the first-order degradation rates of [CIP] and [OFX] = 0.25 mM, pH 7.4 on (a) [Mg(H_2_O)_6_^2+^] at [PDS] = 20 mM, and on (b) [PDS] at [Mg(H_2_O)_6_^2+^] = 2 mM. Measured after approximately 1 h. Error bars represent experimental uncertainty.

Followed by:

2a substrate-OS(O)_3_ + H_2_O → substrate

<svg xmlns="http://www.w3.org/2000/svg" version="1.0" width="13.200000pt" height="16.000000pt" viewBox="0 0 13.200000 16.000000" preserveAspectRatio="xMidYMid meet"><metadata>
Created by potrace 1.16, written by Peter Selinger 2001-2019
</metadata><g transform="translate(1.000000,15.000000) scale(0.017500,-0.017500)" fill="currentColor" stroke="none"><path d="M0 440 l0 -40 320 0 320 0 0 40 0 40 -320 0 -320 0 0 -40z M0 280 l0 -40 320 0 320 0 0 40 0 40 -320 0 -320 0 0 -40z"/></g></svg>


O + H_2_SO_4_

This is clearly a Lewis acid activation mechanism. The following are other plausible mechanisms, which are analogous to oxidations by Fe^IV^=O_aq_,^59–65^

2b HS_2_O_8_^−^/H_2_O)_4_Mg-OS(O)_2_OOS(O)_3_ + substrate → Mg(H_2_O)_6_^2+^ + (substrate-OSO_3_˙^−^ + SO_4_˙^−^)

2c Mg(H_2_O)_6_^2+^ + (substrate-H˙ + SO_4_˙^−^ + SO_4_^2−^

2d Mg(H_2_O)_6_^2+^ + substrate-H^−^ + 2SO_4_^2−^

Reactions (2b) and (2c) differ from the oxidations by Fe^IV^=O^2+^_aq_ by one major aspect: here the products of reactions (2b) and (2c) consist of two radicals that are formed in proximity and are therefore expected to react with each other. Reaction (2a) is analogous to the reaction of Fe^IV^=O^2+^_aq_.^66^ However, other oxygen atom transfer reactions, *e.g.*, epoxidations, are plausible.^67^

It is also proposed that the acidic hydrolysis of S_2_O_8_^2−^

3 S_2_O_8_^2−^ + H_3_O^+^ → SO_4_^2−^ + HSO_5_^−^ + 2H^+^

proceeds *via* an analogous process where the substrate H_2_O reacts with HS_2_O_8_^−^, see DFT below, and not *via* H^+^ binding to an oxygen of the SOOS group^68^ or formation of a SO_4_ (ref. 69) intermediate as earlier proposed.

Despite the superior kinetics observed under acidic conditions, operation near neutral pH (7.0–7.4) is of greater environmental relevance, as most natural and engineered water systems exist within this range. Fig. 3(a) and (b) present the dependence of the degradation kinetics of OFX and CIP on [Mg(H_2_O)_6_^2+^] and on [PDS]. Notably, CIP consistently exhibited higher reactivity than OFX. The slight deviations from the expected linear dependencies in the concentrations are attributed to the fact that the ionic strengths, which do not depend linearly on the concentrations, affect the equilibrium constant of reaction (1) and the ratio [antibiotic-H^+^]/[antibiotic-zwitterion] differently.

To understand the mechanism of the degradation process, the mechanism of the reaction of (H_2_O)_4_Mg(OS(O)_2_OOS(O)_3_) with simple molecules was studied. First, dimethyl sulfoxide (DMSO) was used as a diagnostic probe; the results are presented in Fig. 4.

**Fig. 4:**
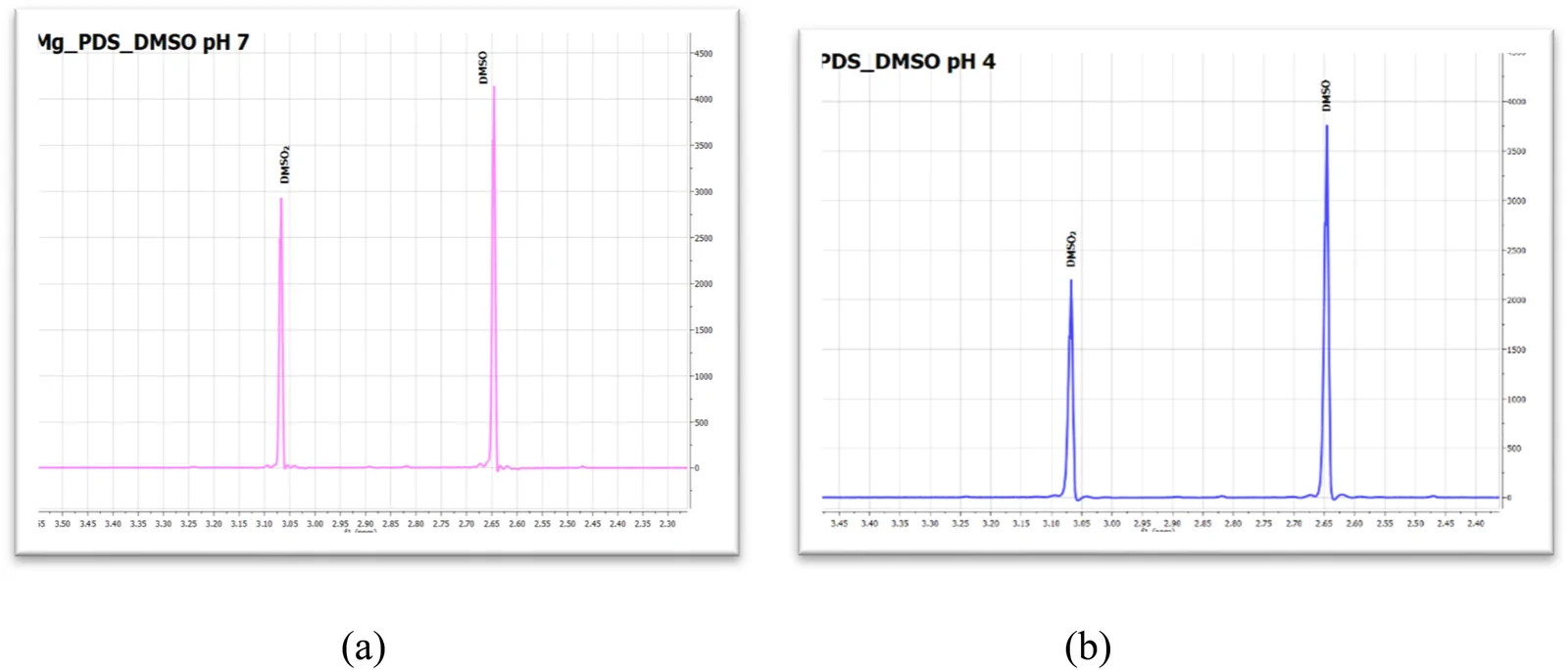
^1^H NMR spectra of dimethyl sulfoxide (DMSO) oxidation under different reaction conditions. (a) At pH 7.0, spectra were recorded after 24 h for the Mg^2+^/PDS/DMSO system. (b) At pH 4.0, spectra were recorded after 4 h for the PDS/DMSO system. Experimental conditions: [PDS] = 100.0 mM, [Mg^2+^] = 50.0 mM, and [DMSO] = 20.0 mM.

The appearance of dimethyl sulfone signals confirms oxidation *via* oxygen atom transfer, according to the Lewis acid activation mechanism. The results, Fig. 4, show that the major product of this process is dimethyl sulfone (DMSO_2_), *i.e.* the process observed does not involve SO_4_˙^−^ radical anions, or Cl˙/Cl_2_˙^−^, formation. It is proposed that the reactions occurring are reactions −5 in analogy to the reaction of PMS with DMSO^70^

4 Mg(H_2_O)_6_^2+^ + S_2_O_8_^2−^ ⇌ (H_2_O)_4_Mg(OS(O)_2_OOS(O)_3_) + 2H_2_O

5 (H_2_O)_4_Mg(OS(O)_2_OOS(O)_3_) + (CH_3_)_2_SO → (H_2_O)_4_Mg(OS(O)_2_OOS(O)_2_⋯S(O)(CH_3_)_2_ + H_2_O → (H_2_O)_5_Mg-OS(O)_3_ + SO_3_ + (CH_3_)_2_SO_2_

In our previous study of the Mg^2+^/H_2_O_2_/HCO_3_^−^ system, the transient (H_2_O)_5_Mg-OC(O)OOH^+^ complex was proposed to undergo homolytic O–O bond cleavage, generating radical intermediates. This interpretation was based on the experimental evidence available at that time and on the mechanistic framework commonly applied to Fenton-like peroxide activation. The present observation that Lewis-acid activation of PDS can initiate oxidations *via* reaction (4) prompted us to re-examine the earlier Mg^2+^/H_2_O_2_/HCO_3_^−^ system using DMSO as a mechanistic probe. As shown in Fig. 5, DMSO_2_ was observed as the oxidation product. This new evidence indicates that the initiating step is more appropriately described as Lewis-acid-induced heterolytic O–O bond activation rather than the previously proposed homolytic cleavage. Thus, the present results refine our earlier mechanistic interpretation based on new experimental evidence specifically probing the mode of O–O bond cleavage. This conclusion concerns the initiating activation step and does not exclude subsequent radical pathways for other substrates.

**Fig. 5:**
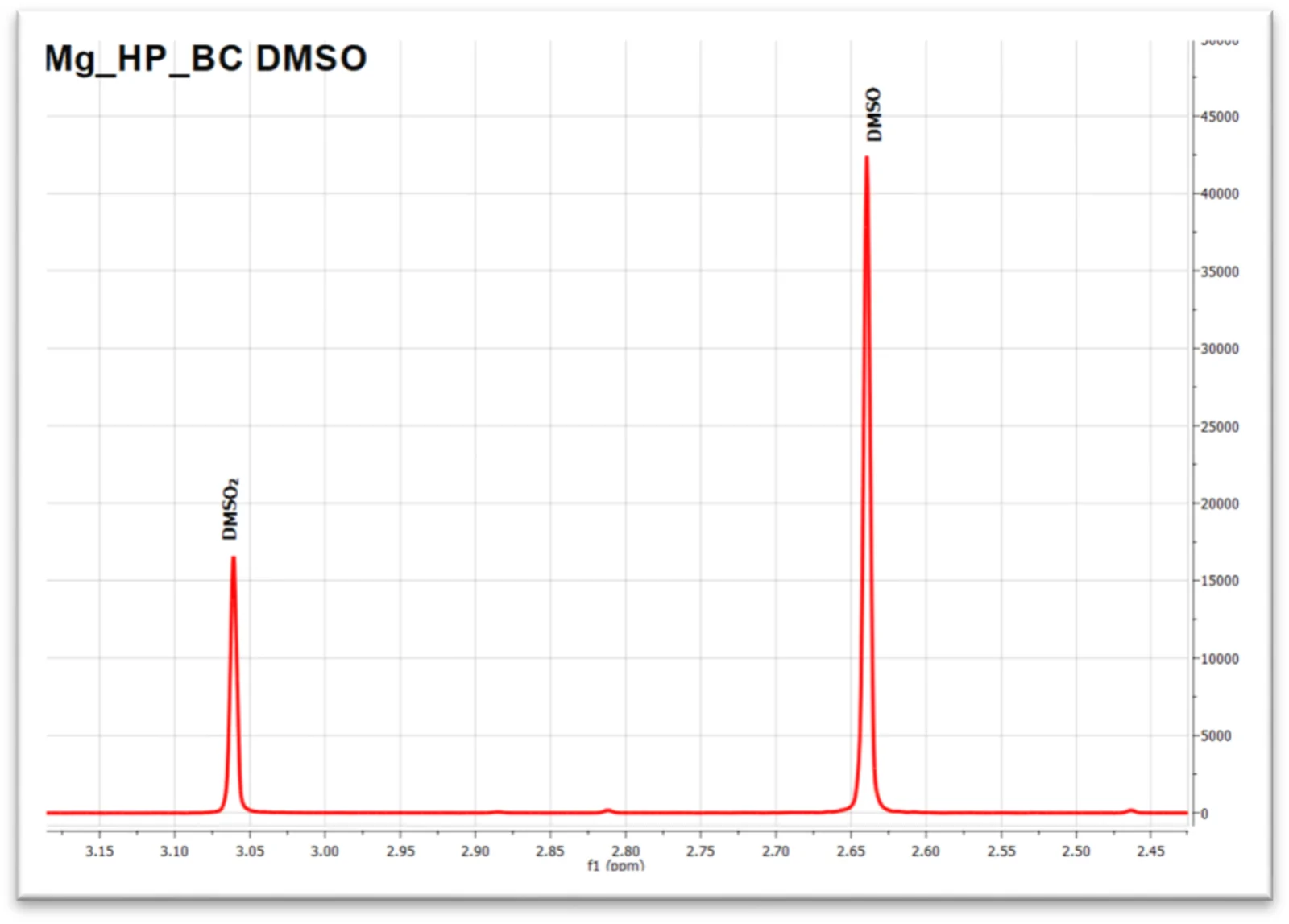
^1^H NMR spectrum of the products of the reactions of Mg^2+^ + H_2_O_2_ + HCO_3_^−^ with DMSO at pH 7.4 measured 3 h after preparation of the sample. Experimental conditions: [H_2_O_2_] = 100.0 mM, [HCO_3_^−^] = 100.0 mM, [Mg^2+^] = 2.0 mM, and [DMSO] = 20.0 mM, de-aerated solutions.

As CIP and OFX are aromatic molecules, phenol was additionally employed as a model substrate to examine the reactivity of the Mg^2+^/PDS system. As shown in Fig. 6, Mg^2+^ alone resulted in negligible phenol degradation at both pH 4 and 7. At pH 4, PDS alone resulted in 20% and 45% degradation after 4 and 24 h, respectively, whereas the Mg^2+^/PDS system increased the degradation to 28% and 56%. At pH 7, PDS alone resulted in 10% and 14% degradation, while Mg^2+^/PDS achieved 18% and 36% degradation after 4 and 24 h, respectively. These results demonstrate that Mg^2+^ enhances PDS-mediated phenol oxidation at both acidic and neutral pH, with the enhancement becoming particularly evident after prolonged reaction at pH 7.

**Fig. 6:**
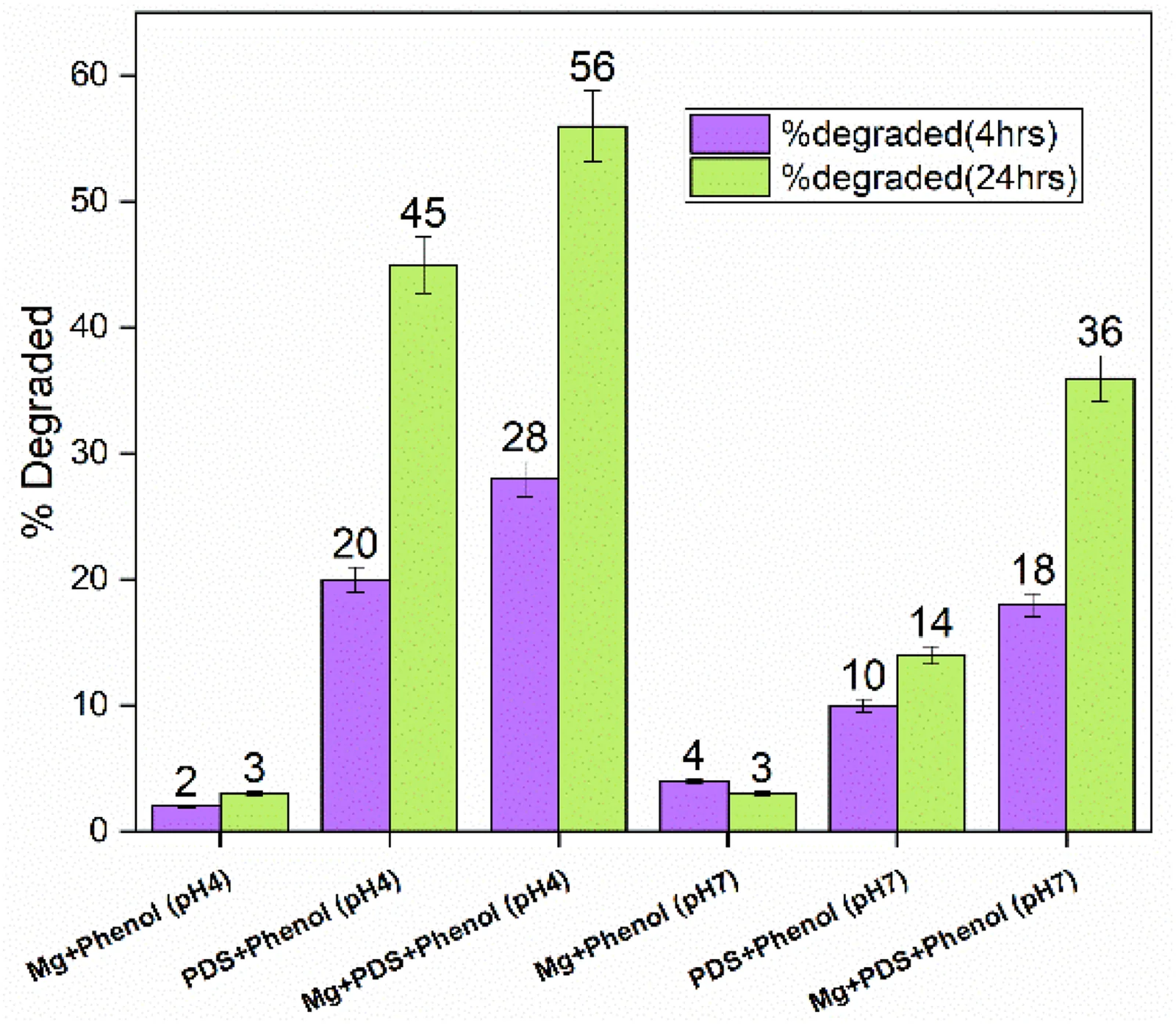
Removal efficiency of phenol for the systems: Mg^2+^ + phenol, PDS + phenol, Mg^2+^ + PDS + phenol. ([PDS] = 10 mM, [MgCl_2_·6H_2_O] = 2.0 mM, [phenol = 0.25 mM]). All samples were at pH 7.40 and pH 4, aerated, and analysed by HPLC after 4 h and 24 h of preparation.

### 3.2 DFT results

To evaluate the plausibility of PDS activation by strong Lewis acids, two processes, in which H^+^ acts as a Lewis acid, were studied using DFT: hydrolysis of PDS and the reaction of PDS with DMSO. The last reaction was also studied with Mg^2+^ as a Lewis acid. In addition, the reaction of (H_2_O)_4_Mg(OS(O)_2_OOS(O)_2_O) with phenol was investigated computationally, suggesting a radical mechanism.

#### 3.2.1 Hydrolysis of HS_2_O_8_^−^

The reaction proceeds in two steps:

6a HS_2_O_8_^−^ + H_2_O → H_2_SO_4_ + HSO_5_^−^ Δ*G*^0^ = 0.78 kcal mol^−1^ Δ*G*^#^ = 43.35 kcal mol^−1^

6b H_2_SO_4_ → HSO_4_^−^ + H^+^ Δ*G*^0^ (pH 0) = −5.62 kcal mol^−1^

6 HS_2_O_8_^−^ + H_2_O → HSO_4_^−^ + HSO_5_^−^ + H^+^ Δ*G*^0^ (pH 0) = −4.84 kcal mol^−1^

In the first step, H_2_O binds to the O–O bond; this bond is cleaved, and two products are formed, HSO_5_^−^ and H_2_SO_4_, in an isoenergetic reaction with a very high barrier; therefore, it proceeds very slowly. Then, H_2_SO_4_ is deprotonated depending on the pH. In the calculation of 6b, the experimental value for the solvation energy of H^+^ (−264 kcal mol^−1^)^71^ was used, as DFT cannot calculate this value. The structures of the reactants (IS), products (FS), and transition state (TS), including the imaginary frequency, are depicted in Table 2.

**Table 2:** Structures of the reactants (IS), products (FS), and transition state (TS). The imaginary frequencies are written under the TS. A broken blue line denotes cleaved bonds

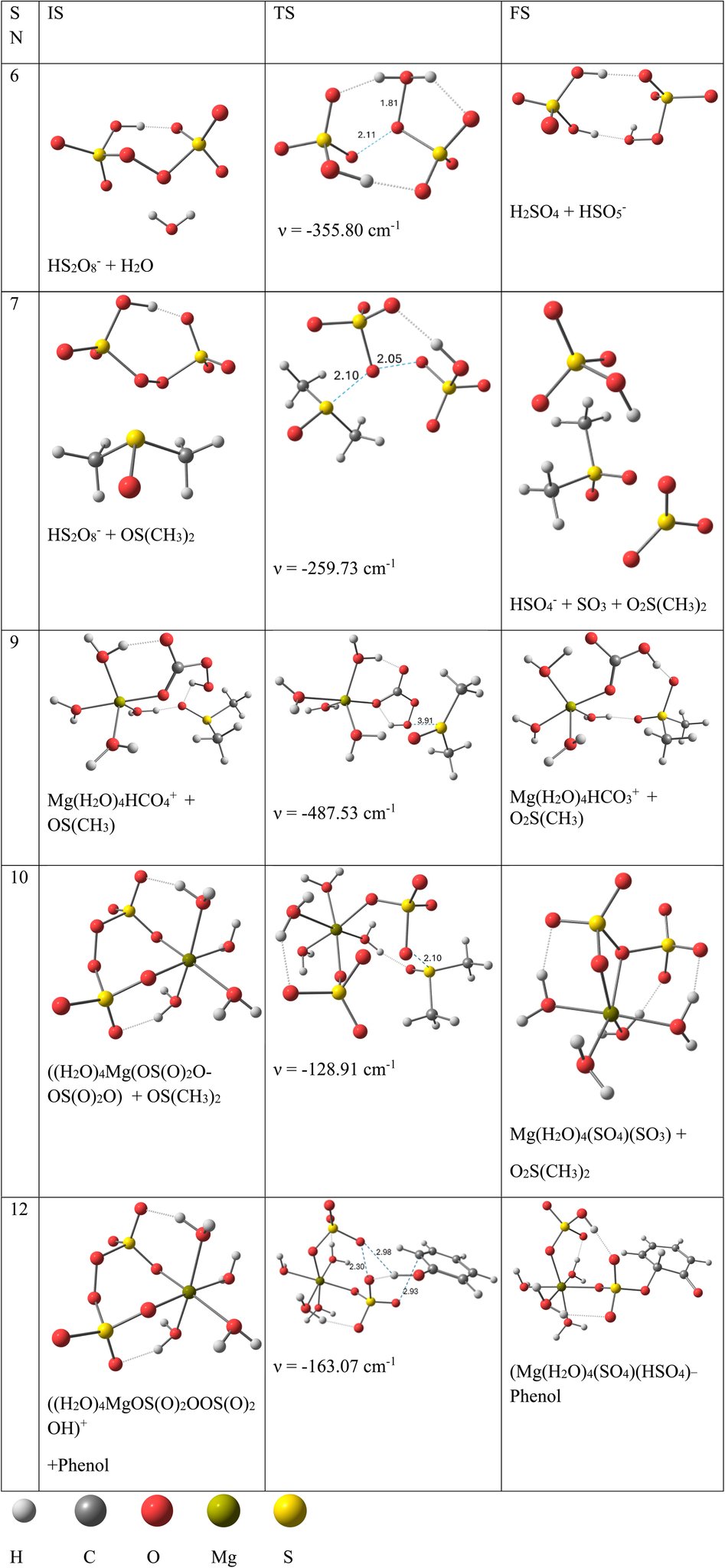

#### 3.2.2 Reaction of PDS with DMSO

This process is described in reaction (7):

7 HS_2_O_8_^−^ + OS(CH_3_)_2_ → SO_3_ + HSO_4_^−^ + O_2_S(CH_3_)_2_ Δ*G*^0^ = −44.01 kcal mol^−1^ Δ*G*^#^ = 5.34 kcal mol^−1^

This is a very exergonic, fast reaction, with a very low barrier, in which the O–O bond is cleaved heterolytically, and one oxygen binds to the sulfur of the DMSO, and SO_3_ + HSO_4_^−^ are formed; then, SO_3_ is hydrolyzed to form HSO_4_^−^/SO_4_^2−^ according to the pH. The structures of this reaction are given in Table 2. The reaction observed is orders of magnitude slower than the calculated value as the concentration of HS_2_O_8_^−^ is clearly very low, the p*K*_a_ of HS_2_O_8_^−^ is unknown.

Recently, we reported that magnesium(ii)-bicarbonate complexes catalyse the Fenton-like reaction under nearly neutral conditions.^38^ The mechanism suggested in that manuscript was based on radical formation, and highly endergonic steps (∼35 kcal mol^−1^) with high barriers (∼35 kcal mol^−1^) were involved. In view of the redox mechanism suggested herein, the mechanism for magnesium(ii)-bicarbonate was re-studied. The studied mechanism was based on the direct reaction of Mg(H_2_O)_4_(HCO_4_)^+^ with DMSO.

The formation of the peroxy-carbonate Mg(H_2_O)_4_(HCO_4_)^+^ is almost an isoenergetic reaction:

8 Mg(H_2_O)_4_(HCO_3_)(H_2_O_2_)^+^ → Mg(H_2_O)_4_HCO_4_^+^ + H_2_O Δ*G*^0^ = 2.24 kcal mol^−1^

Mg(H_2_O)_4_HCO_4_^+^ reacts directly with DMSO:

9 Mg(H_2_O)_4_HCO_4_^+^ + OS(CH_3_)_2_ → Mg(H_2_O)_4_HCO_3_^+^ + O_2_S(CH_3_)_2_ Δ*G*^0^ = −59.09 kcal mol^−1^ Δ*G*^#^ = 38.60 kcal mol^−1^

This is a very slow, highly exergonic reaction. The activation barrier in this mechanism is almost identical to the barrier reported previously.^38^ However, in this mechanism, only one highly exergonic step is involved, which drives the reaction to completion; therefore, this mechanism is more plausible. The structures associated with this reaction are also presented in Table 2.

#### 3.2.3 Reaction of (H_2_O)_4_Mg(OS(O)_2_OOS(O)_2_O) with DMSO

10 (H_2_O)_4_Mg(OS(O)_2_OOS(O)_2_O) + OS(CH_3_)_2_ → (Mg(H_2_O)_4_(SO_4_)(SO_3_))^+^ + O_2_S(CH_3_)_2_ Δ*G*^0^ = −42.01 kcal mol^−1^ Δ*G*^#^ = 17.05 kcal mol^−1^

11 (Mg(H_2_O)_4_(SO_4_)(SO_3_)) → Mg(H_2_O)_3_(HSO_4_)_2_ Δ*G*^0^ = −10.97 kcal mol^−1^

Mg^2+^ binds both sulfates of PDS, forming a bidentate structure with two Mg–O–S bonds.

This structure is depicted in Table 2. The reaction with DMSO follows exactly the mechanism of reaction (7); it proceeds *via* an oxygen transfer. The exergonicity of reaction (10) is similar to that of reaction (7), but it has a higher barrier, which is nevertheless relatively moderate.

#### 3.2.4 Reaction of (H_2_O)_4_Mg(OS(O)_2_OOS(O)_2_O) with phenol

12 (H_2_O)_4_Mg(OS(O)_2_OOS(O)_2_O) + phenol → ((H_2_O)_4_MgOS(O)_2_OOS(O)_2_O)-phenol Δ*G*^0^ = −41.75 kcal mol^−1^ Δ*G*^#^ = 22.75 kcal mol^−1^

The reaction proceeds *via* a radical mechanism initiated by the homolytic cleavage of the O–O bond in PDS, generating two sulfate radicals. One sulfate radical abstracts the hydroxyl hydrogen from phenol through homolytic O–H bond cleavage; concurrently, the resulting phenoxy radical undergoes *ortho* attack by the other sulfate radical, yielding the *ortho*-sulfated phenol product, in this case, no persistent radical intermediates are formed. If occasionally, another substrate (*e.g.*, another phenol molecule) hits the phenoxy radical, radical intermediates, that can lead to polymerization. This is a highly exergonic reaction with a moderate barrier. Thus, the DFT calculations indicate that the reaction of the Mg/PDS complex with the phenol results in the formation of radicals. The degradation of the aromatic CIP and AFX is expected to follow this mechanism.

All the structures are depicted in Table 2.

## 4 Concluding remarks

The results presented herein prove that PDS can be activated by strong Lewis acids to oxidize antibiotics and DMSO. This activation is not initiated by the formation of radicals, as proven by the oxidation of DMSO to form DMSO_2_. However, the results do not rule out radical involvement in the catalytic oxidations of the antibiotics studied. Moreover, the reaction of phenol with PDS, catalysed by Mg^2+^, is a radical process.

This activation contradicts the commonly accepted assumption that peroxides can be activated towards advanced oxidations only *via* two routes: electron transfer (*e.g.*, Fenton-like, electrochemistry) or energy transfer (*e.g.*, heat; photochemistry).^72^

The results prove that the oxidation of sulfoxides to sulfones does not always involve oxidation by intermediates of the type M^*n*+2^=O_aq_ as commonly assumed.^66^ Recently, it was shown, using isotope labeling, that Cu^2+^_aq_ activation of PMS and PDS leads to oxygen transfer from the peroxide to the substrate.^73^ It was proposed that the active species in the system is CuO^+^, however, this proposal is wrong as Cu^III^ in neutral solutions is uncharged.^74^ The results of the EPR study fit the idea that Cu(ii) forms Cu^II^–OS(O)_2_OOH_aq_^+^/Cu^II^–OS(O)_2_OOSO_3aq_ and Lewis acid activates the persulfates to transfer an oxygen atom to the DMPO. All peroxides based on X^*n*^O_*m*_^(*n*−2*m*)−^, (*n* − 2*m*) ≥ 2, are expected to be candidates for Lewis acid activation as demonstrated herein for S_2_O_8_^2−^ and CO_3_^2−^.

This manuscript reports two significant and previously unrecognized findings. First, we demonstrate, for the first time, that Mg^2+^ can effectively catalyse oxidation processes involving peroxy-disulfate (PDS). Second, we uncover a novel reaction mechanism in which Lewis acid activation of PDS promotes oxygen-atom transfer, as demonstrated for DMSO. The results thus open the door to the development of new advanced oxidation processes with the potential to be environmentally compatible and cost-effective; however, further research is required to assess their practical applicability, including mineralization efficiency and the toxicity of transformation products.

## Author contributions

Conceptualization – D. M., Y. A., A. L., H. K.; data curation, investigation, formal analysis – A. L., H. K.; methodology – D. M.; project administration – D. M.; software – H. K.; supervision – D. M, Y. A.; visualization – A. L., H. K.; writing – original draft – A. L.; writing – review and editing – D. M., Y. A., H. K., A. L.

## Conflicts of interest

There are no conflicts to declare.

## Supplementary Material

RA-OLF-D6RA06842A-s001

## Data Availability

Any additional data will be provided upon request. Supplementary information (SI): HPLC chromatograms and information on the materials and the kinetics for the degradation of ciprofloxacin (CIP) and ofloxacin (OFX are given in the SI. In addition, the *xyz* coordinates of all the relevant structures, together with their electronic energies, are provided. See DOI: https://doi.org/10.1039/d6ra06842a.
