# Supplementary material for: Lewis-acid activation of persulfate enables oxidation of antibiotics: a new green low-cost advanced oxidation process
Source: RSC Adv. 2026 Oct 21;16(48):64986–97. doi: 10.1039/d6ra06842a (PMC13684428; doi:10.1039/d6ra06842a)
Supplement: Supplementary file 1 [file RA-016-D6RA06842A-s001.pdf]

Supplementary Information (SI) for RSC Advances.
This journal is © The Royal Society of Chemistry 2026
Supplementary Information (SI) for RSC Advances.
This journal is © The Royal Society of Chemistry

SUPPLEMENTARY INFORMATION

Lewis-Acid Activation of Persulfate Enables Oxidation of Antibiotics: A New Green Low-Cost Advanced Oxidation Process

Anushka Lucas[1], Haya Kornweitz[1*], Yael Albo[2*] and Dan Meyerstein[1*]

[1] Chemical Sciences Department and The Radical Research Center, Ariel University, Ariel, Israel

[2]Cemical Engineering Department and The Radical Research Center, Ariel University, Ariel, Israel

[*] Corresponding author

Materials

Materials: The chemicals were of analytical grade and were used without further purification and are as follows:

Compound	CAS No.	Supplier	Purity
Magnesium Chloride Hexahydrate	7791-18-6	Sigma Aldrich	99
Magnesium Sulphate Heptahydrate	10034-99-8	Sigma Chemical	99
Sodium Peroxydisulfate	7775-27-1	Thermo scientific	98
Dimethyl sulfoxide	67-68-5	TCI	99
Ciprofloxacin	85721-33-1	Glentham	98
Ofloxacin	82419-36-1	Glentham	98.5
Sodium hydroxide	1310-73-2	Merck	97
Perchloric acid	7601-90-3	Fluka	60

Water, which was purified by the Millipore System with a resistivity > 15 MΩ.cm, was used in all the experiments

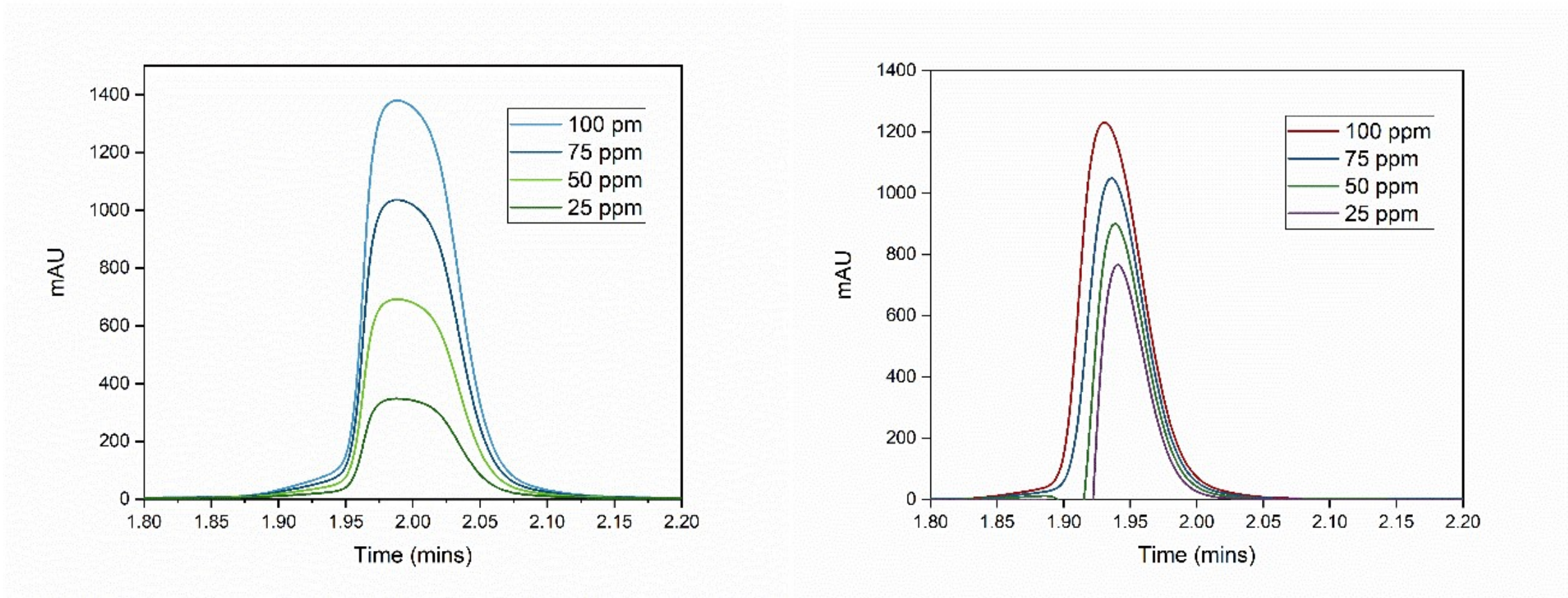

(a) (b)

Figure S1: Representative HPLC chromatograms of calibration standards of ciprofloxacin (a) (CIP) and (b) ofloxacin (OFX) at different concentrations.

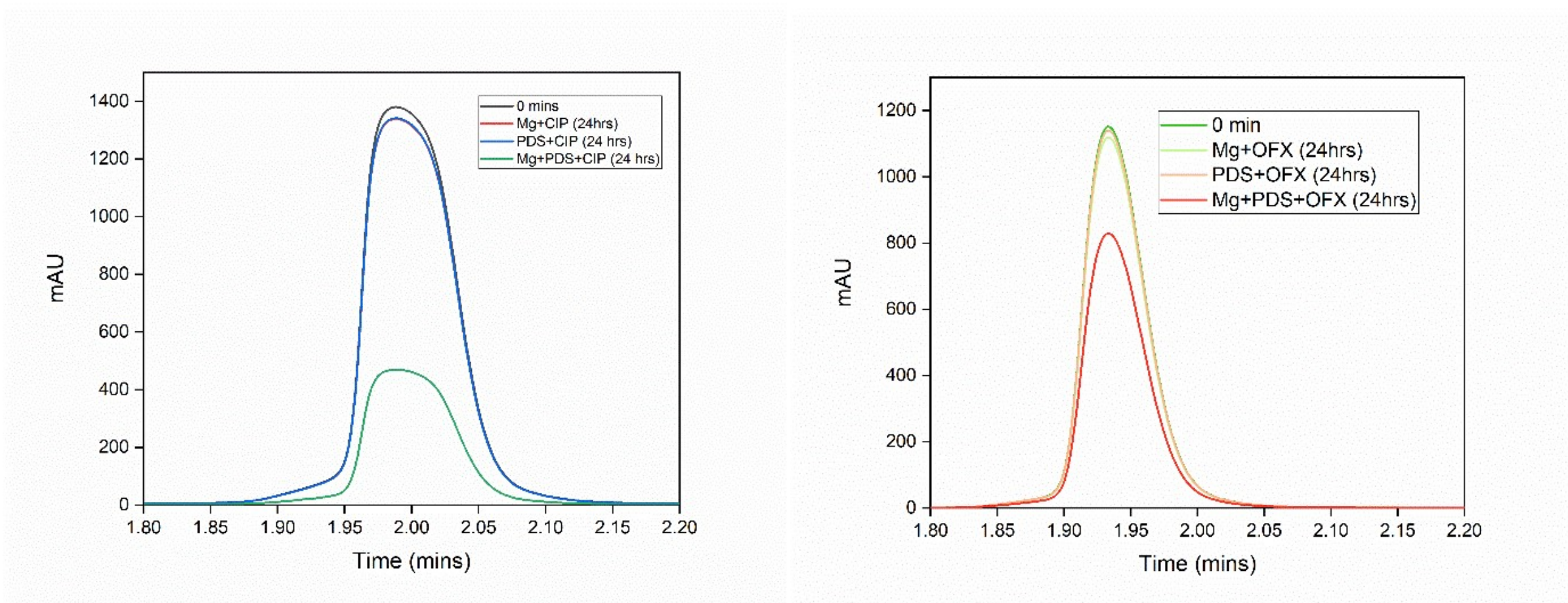

(a) (b)

Figure S2: Representative HPLC chromatograms showing the degradation profiles of (a) ciprofloxacin (CIP) and (b) ofloxacin (OFX) for samples: Mg^{2+}+CIP, PDS+CIP, Mg^{2+}+PDS+CIP and Mg^{2+}+OFX, PDS+OFX, Mg^{2+}+PDS+OFX. ([PDS] = 20 mM, [$MgCl_2{\cdot}6H_2O$] = 2.0 mM, [CIP], [OFX] = 0.25 mM). All samples were at pH 7.40, aerated, and analysed by HPLC after 24 hrs of preparation.

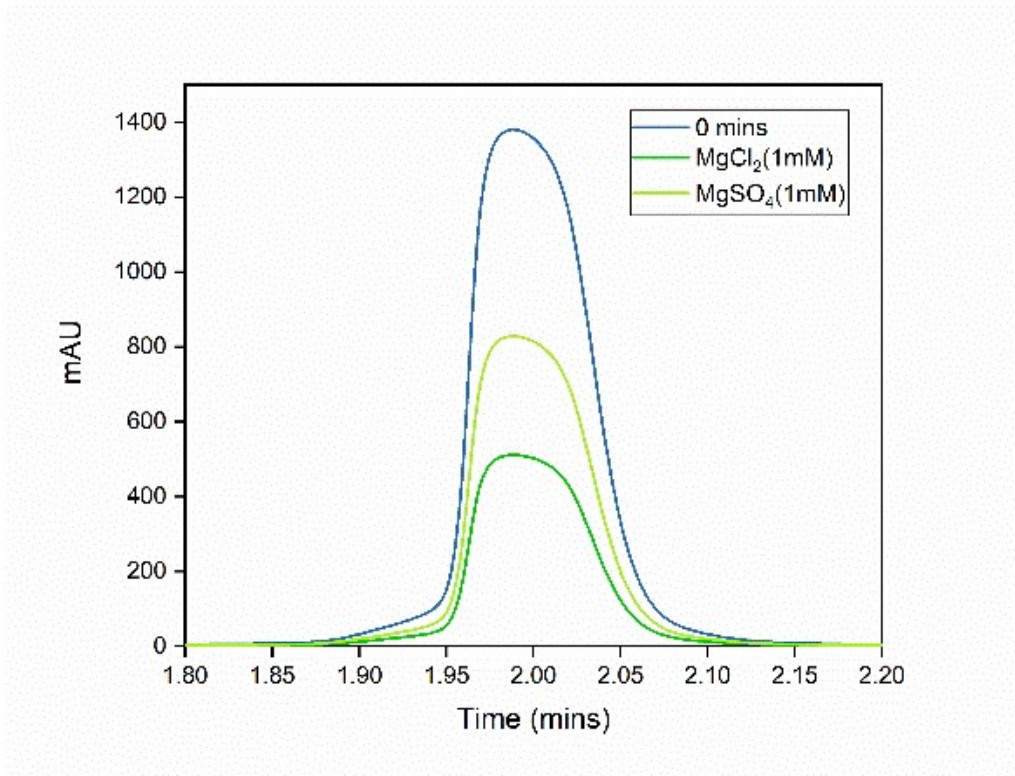

(a)

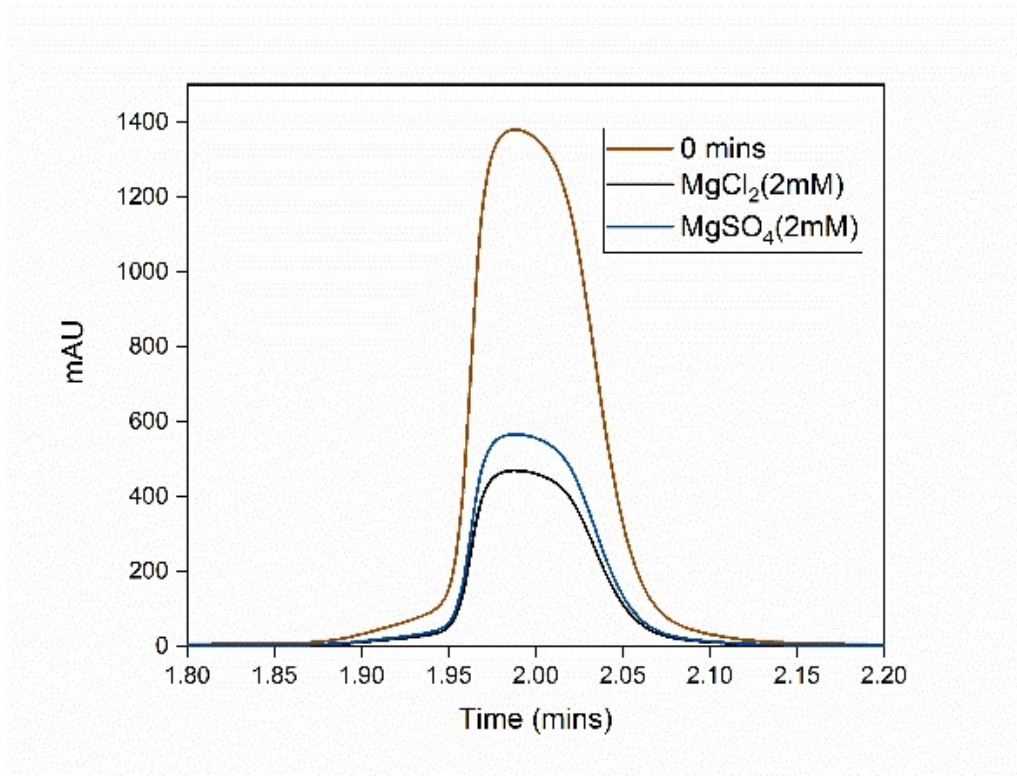

(b)

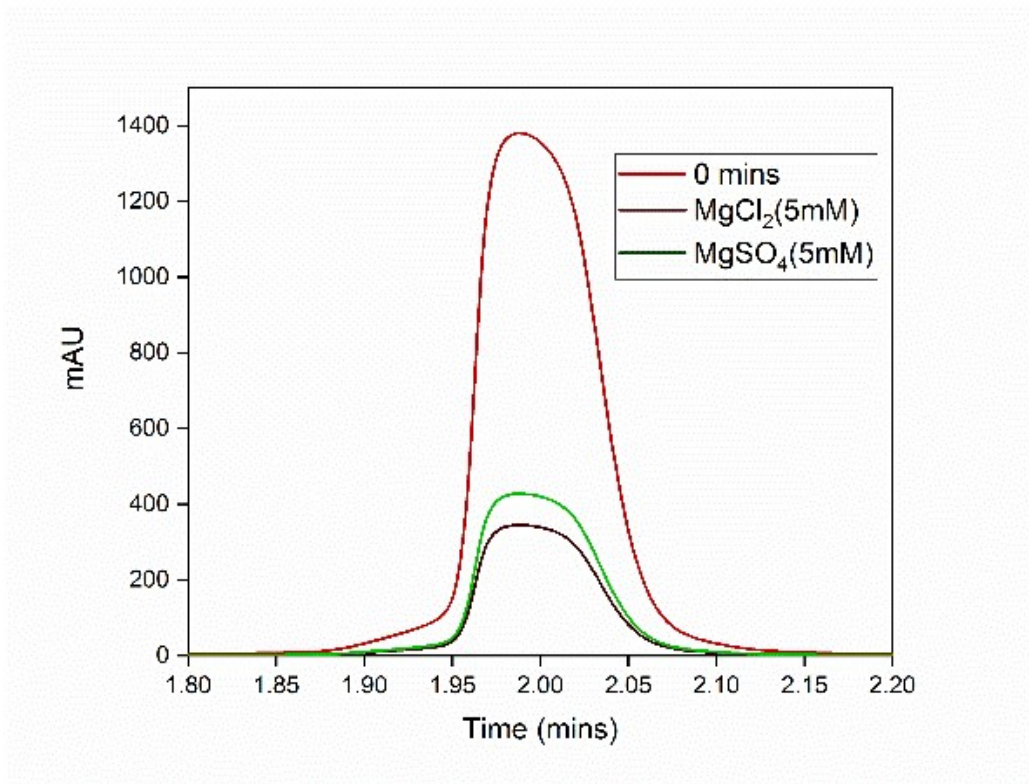

(c)

Figure S3: HPLC chromatograms showing the degradation of ciprofloxacin (CIP) at different concentrations of $MgSO_4$ and $MgCl_2$ (a) 1mM, (b) 2mM, (c) 5mM in aerated solutions by [PDS] = 20 mM of (a) [CIP] = 0.25 mM, pH 7.4

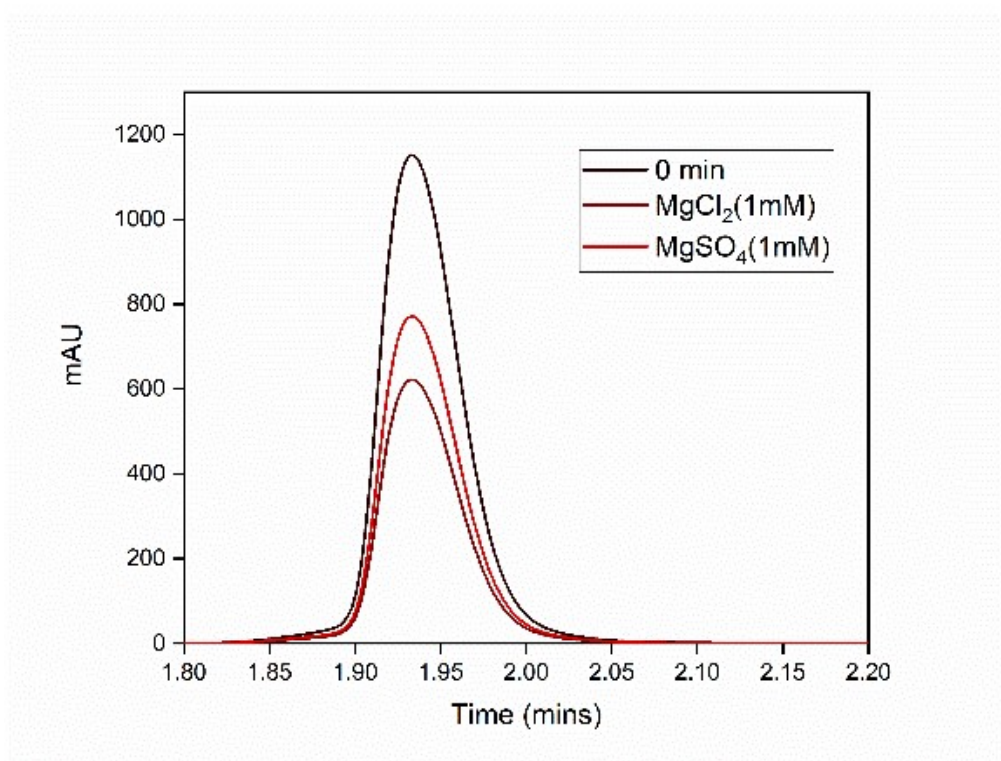

(a)

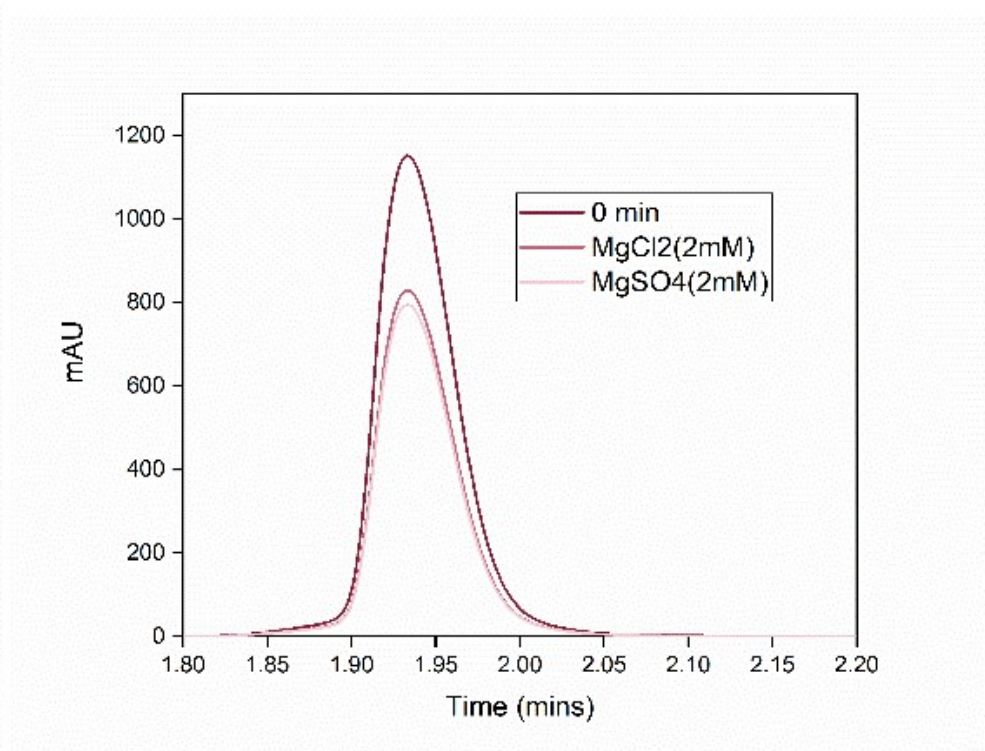

(b)

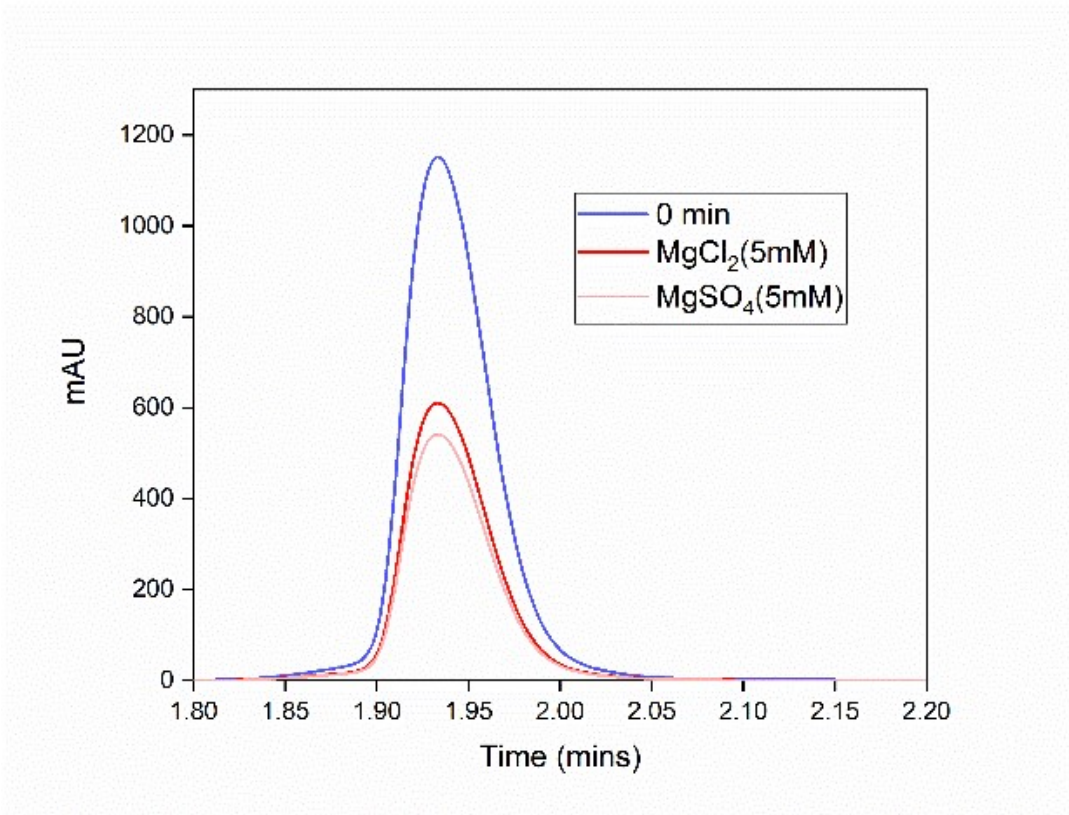

(c)

Figure S4: HPLC chromatograms showing the degradation of ofloxacin (OFX) at different concentrations of $MgSO_4$ and $MgCl_2$ (a) 1mM, (b) 2mM, (c) 5mM in aerated solutions by [PDS] = 20 mM of (a) [CIP] = 0.25 mM, pH 7.4

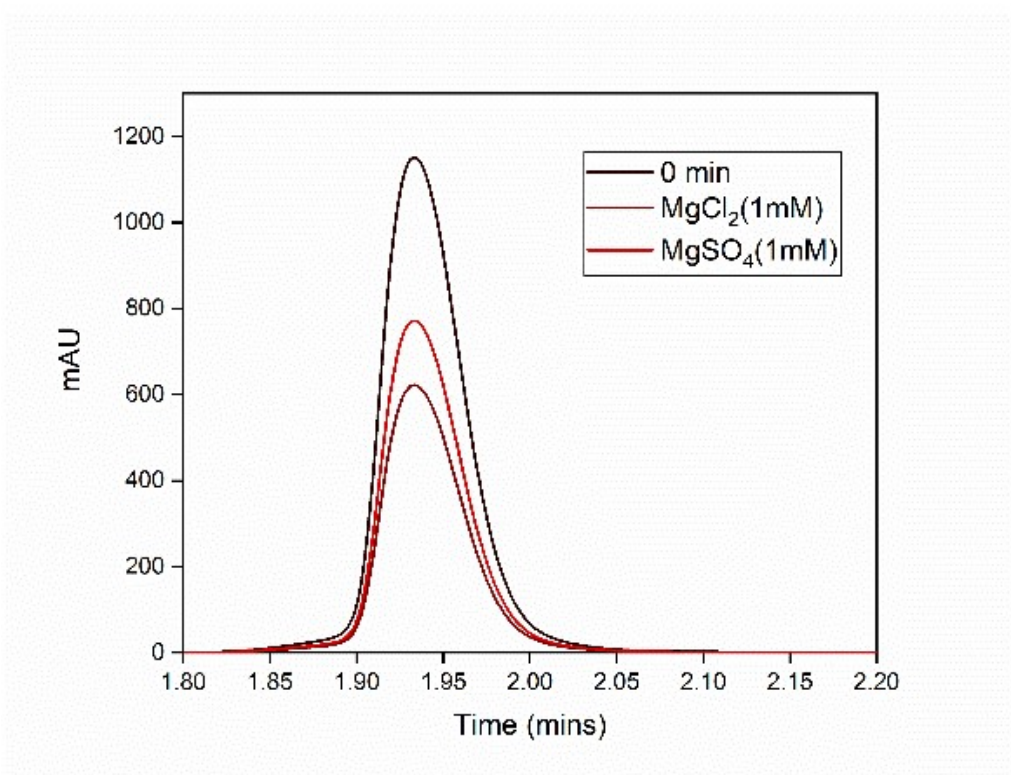

(a)

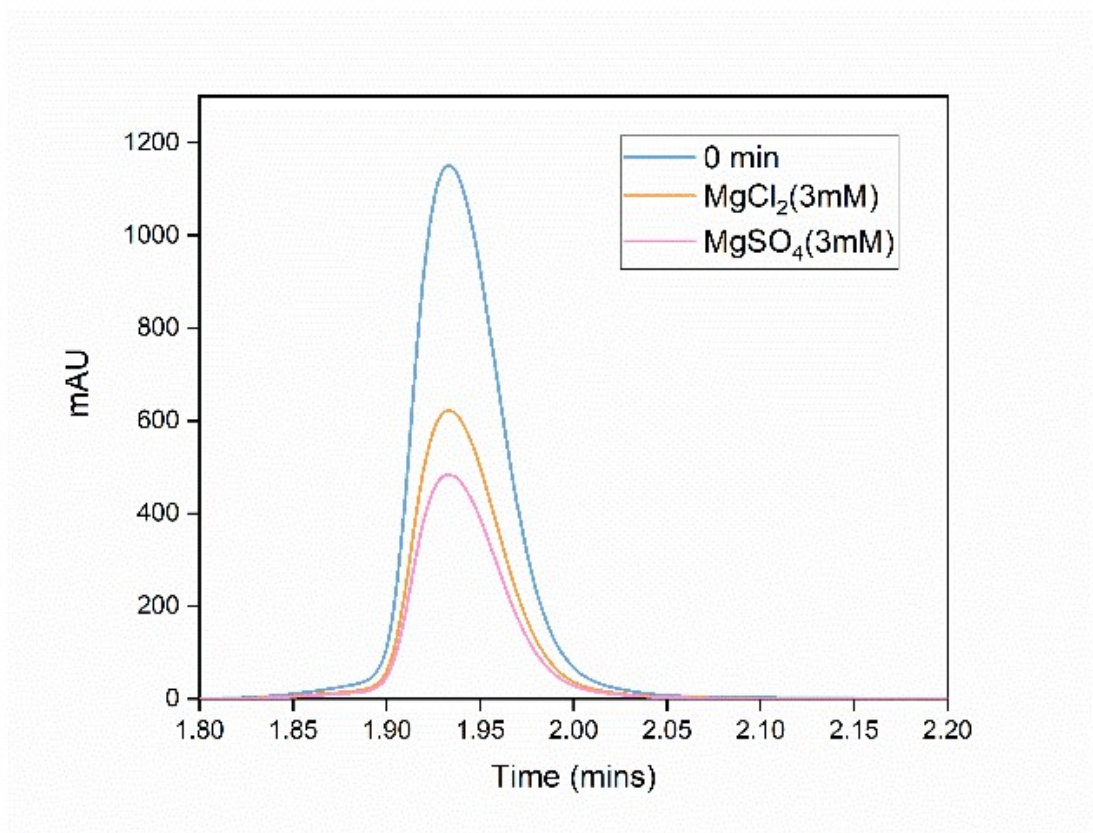

(b)

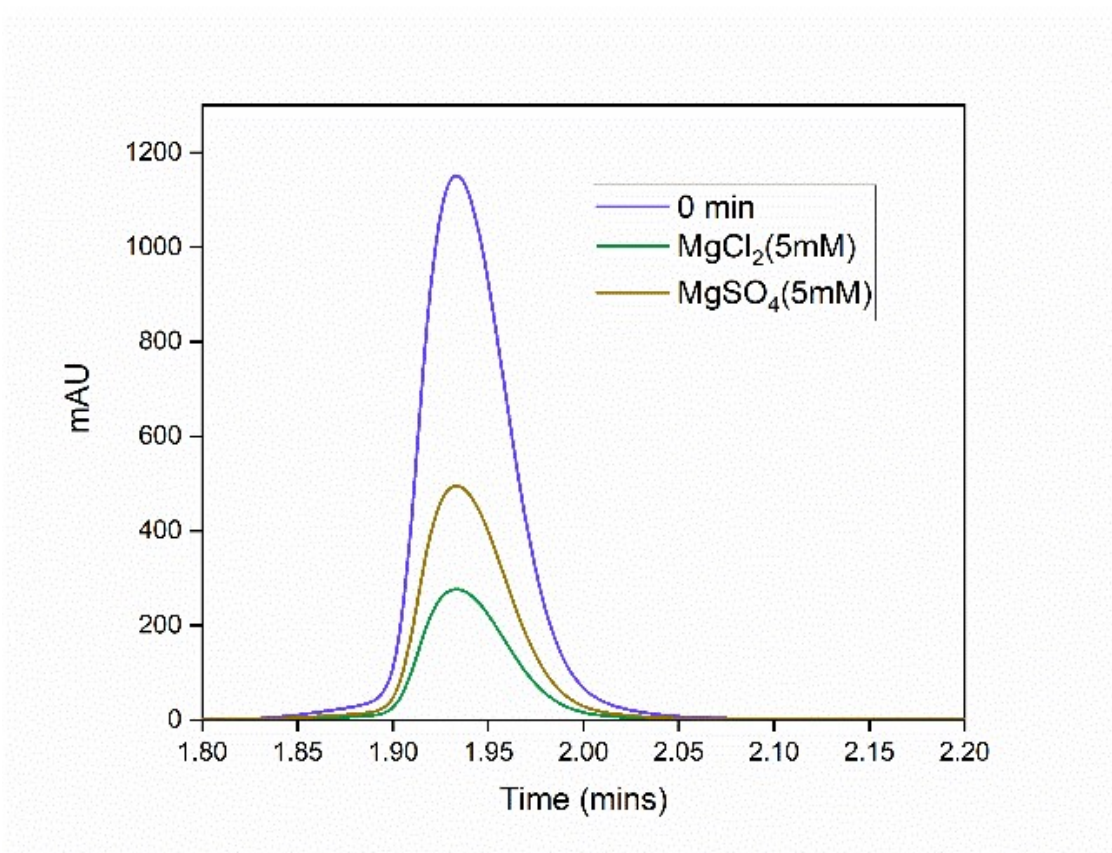

(c)

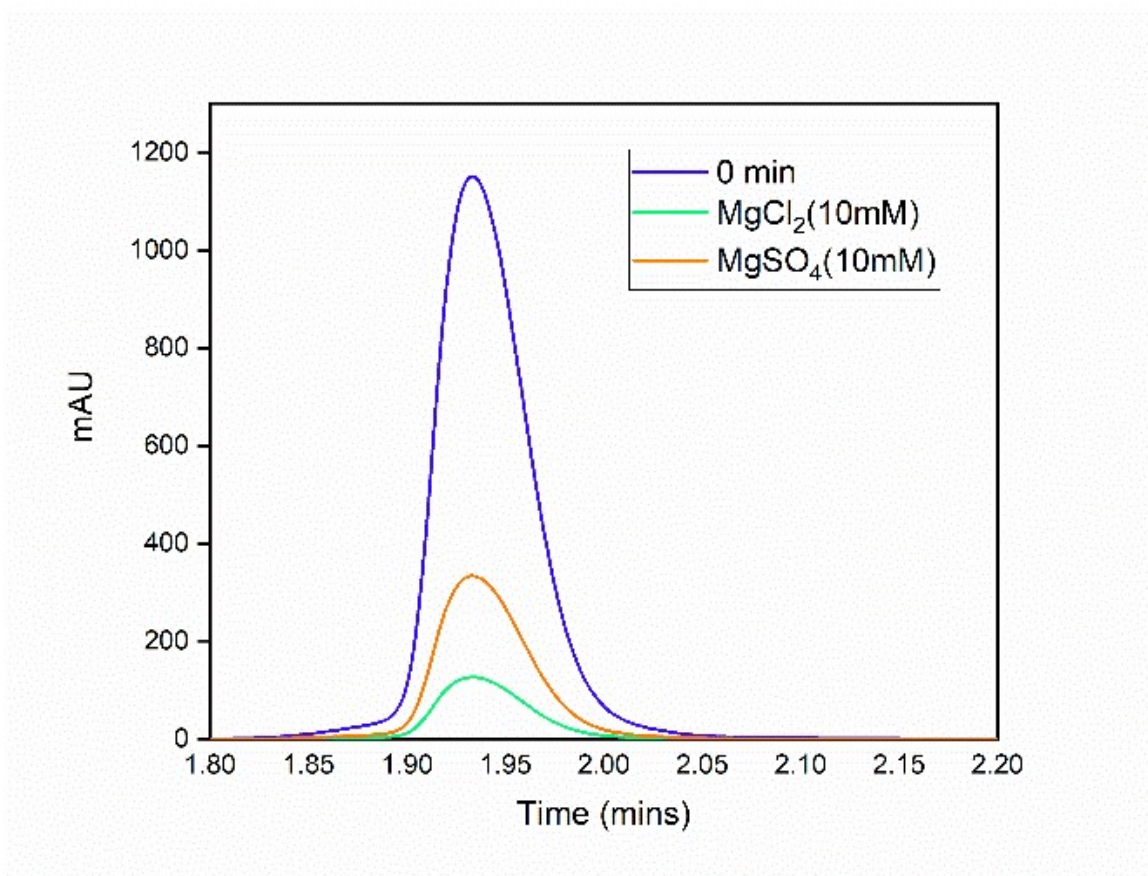

(d)

Figure S5: HPLC chromatograms showing the degradation of ofloxacin (OFX) at different concentrations of $MgSO_4$ and $MgCl_2$ (a) 1mM, (b) 3mM, (c) 5mM (d) 10mM in aerated solutions by [PDS] = 20 mM of (a) [CIP] = 0.25 mM, pH 8.5

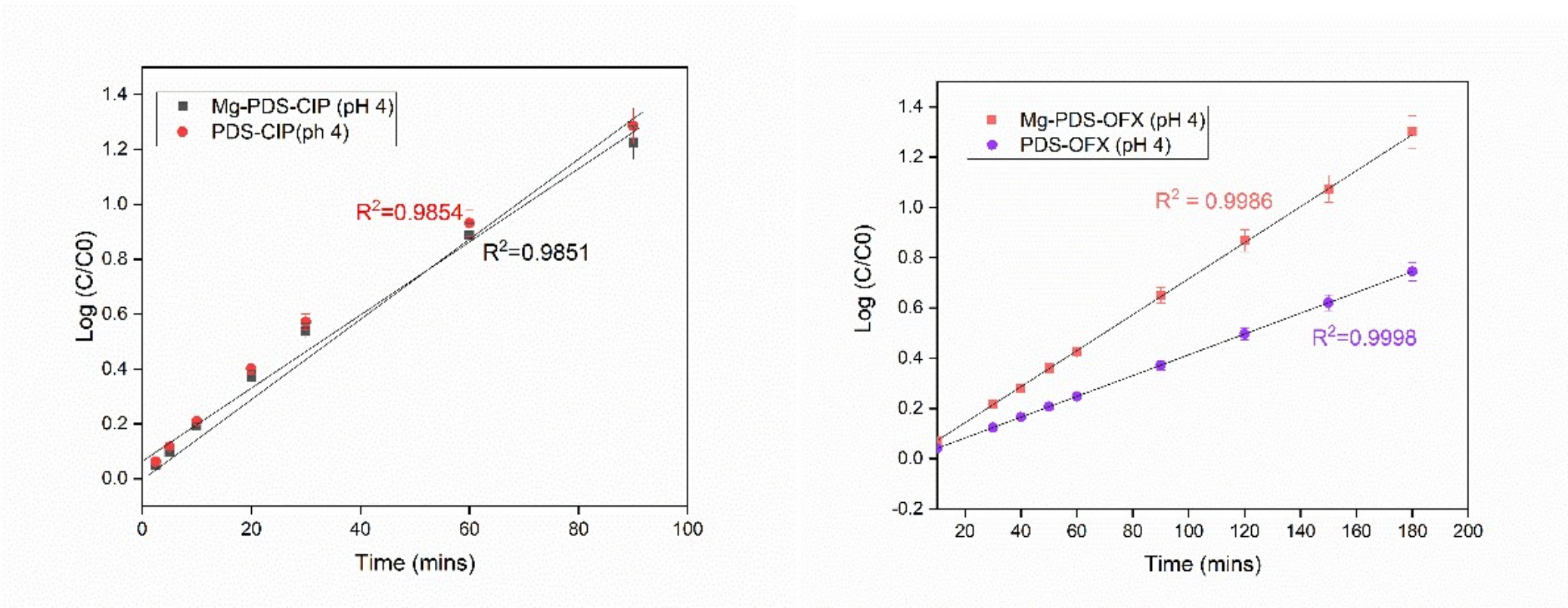

(a) (b)

Figure S6: Pseudo-first-order kinetics for the degradation of Ciprofloxacin (CIP) and Ofloxacin (OFX) (a) Conditions: [CIP] = 0.25mM, [PDS] = 20mM, [Mg] = 2mM pH 4, (b) Conditions: [OFX] = 0.25mM, [PDS] = 20mM, [Mg] = 2mM pH 4.0

Coordinates of the computed structures; the electronic energy in Hartree/particle is given in brackets):

$HS_2O_8^-$ (-1399.00629139):

8	-0.370946000	1.325316000	4.061806000
16	-0.635534000	2.089513000	5.274195000
8	-0.278904000	1.501739000	6.558748000
8	-1.953178000	2.805398000	5.266354000
8	0.512956000	3.424993000	5.279653000
8	0.421176000	4.176332000	4.044493000
16	-0.473638000	5.590275000	4.243825000
8	-0.186754000	6.320468000	3.028625000
8	-1.959355000	5.064923000	4.167947000
8	-0.184573000	6.161881000	5.540865000
1	-2.048337000	4.148711000	4.642953000

HSO5_HSO4 (-1475.36988414):

8	0.308267000	0.917207000	5.202012000
16	0.637454000	2.089951000	6.334168000
8	0.429634000	0.815962000	7.712443000
8	-0.691402000	2.762772000	6.649225000
8	1.129452000	3.266694000	4.897103000
8	0.091990000	3.486024000	3.900725000
16	-0.561168000	4.996285000	4.050423000
8	-1.604266000	5.004514000	3.039776000
8	-1.206664000	4.954910000	5.499309000
8	0.462041000	6.023057000	4.022141000
1	-1.022155000	4.063502000	5.975254000
8	-0.893205000	0.218100000	7.612871000
1	-1.450823000	1.015257000	7.672068000
1	-0.216339000	0.246235000	5.677152000

$Mg(H_2O)_4HCO_4^+$ (-845.704076493):

8	2.114915000	1.065025000	1.308592000
12	0.782824000	0.034541000	0.090999000
8	0.887700000	-1.592797000	1.441255000
8	1.022710000	1.771536000	-1.136688000
8	-1.897370000	-0.863624000	-0.572611000
8	1.979089000	-0.877360000	-1.414377000
8	-0.524360000	-1.273261000	-0.745407000
1	2.377549000	1.961374000	1.066099000
1	2.242703000	0.968389000	2.259128000
1	0.294095000	2.403061000	-1.187904000
1	1.422618000	1.713949000	-2.012876000
6	-1.998941000	0.246375000	0.131951000
1	2.829372000	-1.330620000	-1.399656000

1	1.353341000	-1.432093000	-1.908042000
1	1.321846000	-2.064115000	2.158917000
1	0.231963000	-2.172358000	1.015425000
8	-3.241262000	0.645348000	0.318282000
8	-1.042486000	0.879004000	0.578573000
1	-3.865229000	0.028709000	-0.095727000

$Mg(H_2O)_4HCO_3^{2-}$ (-770.565821054):

8	-1.934287000	-0.441605000	-1.738739000
12	-0.910778000	-0.035907000	0.002651000
8	-1.974872000	-0.219814000	1.757332000
8	-1.356653000	2.013113000	-0.082378000
8	0.881318000	0.741148000	-0.021628000
8	-0.179638000	-1.911961000	0.061402000
1	-2.344973000	0.165822000	-2.365055000
1	-1.784156000	-1.279242000	-2.195017000
1	-2.019223000	2.703509000	-0.182888000
1	-0.470438000	2.412985000	-0.105380000
6	2.010656000	0.139897000	0.001016000
1	0.857371000	-1.750675000	0.061075000
1	-0.379489000	-2.843121000	0.183238000
1	-2.333098000	0.504869000	2.283817000
1	-1.918443000	-0.996328000	2.327730000
8	2.167534000	-1.098030000	0.041006000
8	3.070181000	0.950750000	-0.022296000
1	3.869178000	0.404887000	-0.003026000

$((H_2O)_4MgOS(O)_2OOS(O)_2O)^+$ (-1904.48945968):

12	-0.562198000	0.565193000	0.712560000

8	-2.358233000	-0.492125000	0.381873000
8	-0.599168000	1.188815000	-1.315263000
8	1.127626000	1.607846000	1.076189000
8	-1.503956000	2.320997000	1.442246000
8	0.638144000	-0.998390000	-0.088248000
1	0.003171000	0.597854000	-1.787627000
1	-0.336704000	2.089232000	-1.538412000
16	2.392507000	1.102952000	1.724274000
1	-1.754036000	2.147279000	2.381461000
1	-0.799467000	2.979348000	1.515719000
1	0.408624000	-1.906952000	0.134178000
1	1.563916000	-0.853220000	0.242633000
1	-2.640142000	-0.837362000	1.240369000
1	-3.136759000	-0.196291000	-0.100534000
8	3.319050000	2.149683000	2.078735000
8	2.905235000	-0.089348000	1.020736000
8	1.843615000	0.346710000	3.166072000
16	-0.641555000	0.258382000	3.860639000
8	-1.591391000	1.377483000	4.002393000
8	0.818106000	1.160010000	3.777699000
8	-0.535695000	-0.703907000	4.929405000
8	-0.747031000	-0.371404000	2.491613000

$OS(CH_3)_2$ (-553.298385888):

16	-4.705226000	0.988140000	-4.548385000
8	-3.242473000	0.992022000	-4.938338000
6	-4.935332000	2.351345000	-3.344504000
1	-5.950343000	2.318977000	-2.944548000
1	-4.191167000	2.252322000	-2.553073000

1	-4.777169000	3.280433000	-3.890713000
6	-4.928763000	-0.378401000	-3.347052000
1	-5.943921000	-0.351654000	-2.947053000
1	-4.766146000	-1.305693000	-3.895004000
1	-4.185088000	-0.277301000	-2.555424000

$O_2S(CH_3)_2$ (-628.539638408):

16	1.005823000	1.031959000	-0.926123000
8	1.020301000	-0.251962000	-0.217865000
8	1.626465000	1.160619000	-2.248415000
6	-0.713098000	1.567738000	-1.066721000
1	-1.195922000	0.839354000	-1.717542000
1	-1.172306000	1.556815000	-0.079375000
1	-0.742378000	2.557957000	-1.518875000
6	1.749312000	2.270603000	0.157632000
1	2.793367000	1.978190000	0.265579000
1	1.676675000	3.248705000	-0.315545000
1	1.247263000	2.246952000	1.123670000

Mg(H2O)4(SO4)(SO3) (-1829.31904546):

12	-4.942683000	1.246673000	-0.341053000
8	-7.007314000	0.870224000	-0.616540000
8	-3.879322000	2.243757000	-1.810220000
8	-3.236343000	1.238002000	0.933576000
8	-5.429072000	3.138362000	0.503837000
8	-4.440141000	-0.525456000	-1.312945000
1	-2.945019000	1.945005000	-1.631030000
1	-3.872391000	3.205564000	-1.750333000
16	-1.769850000	0.402734000	0.342279000

1	-6.332806000	3.467707000	0.541195000
1	-5.104656000	3.100486000	1.436623000
1	-4.131075000	-0.495957000	-2.225155000
1	-3.688410000	-0.932551000	-0.798330000
1	-7.192226000	0.475576000	0.251407000
1	-7.319293000	0.238636000	-1.274677000
8	-0.761970000	0.593386000	1.352578000
8	-1.641350000	1.184703000	-0.910934000
8	-2.272410000	-0.970077000	0.122856000
16	-4.198335000	0.831840000	2.311197000
8	-4.470446000	2.165876000	2.869839000
8	-3.527998000	-0.150015000	3.119998000
8	-5.379502000	0.336804000	1.493931000

(Mg(H_2O)$_4$(SO4)(HSO_4)_Phenol (-2212.16182403):

12	-4.655578000	1.307157000	-0.873155000
8	-6.385094000	0.810781000	-2.014984000
8	-4.029030000	2.818268000	-2.228050000
8	-3.105283000	1.992515000	0.228985000
8	-5.896081000	2.613682000	0.218094000
8	-3.394728000	-0.048031000	-1.936173000
1	-3.961962000	2.678090000	-3.178941000
1	-3.163750000	3.119986000	-1.917120000
16	-1.788844000	1.289425000	0.451917000
1	-5.880207000	2.155374000	1.106092000
1	-5.591114000	3.513958000	0.368858000
1	-3.507174000	-0.933419000	-1.565968000
1	-2.447141000	0.170719000	-1.829836000
1	-6.642354000	-0.004098000	-1.554917000

1	-7.107784000	1.432246000	-1.863460000
8	-0.924448000	2.350186000	1.311472000
8	-1.073803000	1.115195000	-0.817056000
8	-1.946931000	0.076893000	1.276238000
16	-5.478627000	-0.391912000	1.693112000
8	-5.507100000	0.997090000	2.237464000
8	-4.164209000	-1.105385000	2.275959000
8	-6.589148000	-1.251665000	2.033138000
8	-5.235627000	-0.302093000	0.185063000
6	-1.390313000	3.767393000	5.159792000
6	-2.544340000	3.230537000	4.453235000
6	-0.140488000	3.710682000	4.654250000
1	-3.527752000	3.361552000	4.888537000
1	0.704750000	4.175508000	5.146859000
6	-2.405899000	2.585749000	3.287225000
6	0.112505000	3.139520000	3.325847000
1	-3.262159000	2.172795000	2.767331000
6	-1.035864000	2.289066000	2.751358000
1	-0.795735000	1.259688000	3.062384000
1	-1.560613000	4.259664000	6.111775000
8	1.156434000	3.285317000	2.727790000
1	-3.350836000	-0.647939000	1.949999000

TS of reaction 6 (-1475.43010794):

8	-0.718822000	0.999612000	3.594338000
16	-0.830355000	2.160405000	4.462955000
8	-0.382882000	1.969927000	5.867810000
8	-2.063544000	2.959443000	4.334184000
8	0.394412000	3.334173000	3.909071000

8	-0.367703000	4.806630000	2.597081000
16	-0.357173000	5.910154000	3.643464000
8	-0.526471000	7.229540000	3.049788000
8	-1.690876000	5.641848000	4.533399000
8	0.795754000	5.815682000	4.586978000
1	-1.915381000	4.678869000	4.491154000
8	1.636935000	3.410147000	5.221535000
1	1.410541000	4.397173000	5.158188000
1	1.035945000	2.983140000	5.886393000

TS of reaction 7 (-1952.31739010)

8	-0.806992000	0.175860000	4.566831000
16	-1.316008000	1.357709000	5.283824000
8	-2.143548000	1.071457000	6.454752000
8	-2.257646000	2.174955000	4.240152000
8	-0.212968000	2.354349000	5.646516000
8	0.804058000	2.478493000	3.870306000
16	0.642749000	4.229910000	3.573060000
8	1.355080000	4.380021000	2.288547000
8	-0.823512000	4.372449000	3.486175000
8	1.299645000	4.850062000	4.719363000
1	-1.784069000	2.992041000	3.943332000
16	2.045233000	1.587459000	2.429632000
6	3.619805000	2.415296000	2.752370000
1	3.652704000	2.632791000	3.818801000
1	4.426768000	1.757874000	2.426922000
1	3.574572000	3.342147000	2.184931000
6	2.059377000	0.099695000	3.442848000
1	2.604856000	-0.671270000	2.899841000

1	2.521901000	0.354212000	4.395059000
1	1.007522000	-0.146072000	3.626256000
8	1.938937000	1.240761000	0.987749000

TS of reaction 9 (-1398.96612547):

8	2.009428000	-2.240968000	0.780649000
12	1.456506000	-0.387400000	0.348537000
8	3.054662000	-0.401057000	-1.039739000
8	2.307721000	1.277328000	1.224939000
8	0.043563000	-0.140402000	1.765150000
8	0.040585000	-0.210933000	-1.073969000
1	2.729376000	-2.528747000	1.349037000
1	1.231855000	-3.022866000	0.838188000
1	3.152631000	1.732358000	1.143735000
1	1.774874000	1.729320000	1.891742000
6	-1.194675000	-0.431310000	1.637839000
1	-0.858254000	-0.266946000	-0.615445000
1	-0.050683000	-0.348794000	-2.020012000
1	3.313760000	0.155973000	-1.782443000
1	3.399498000	-1.289460000	-1.199356000
8	-1.858220000	-0.495911000	0.598878000
8	-1.879469000	-0.712640000	2.780030000
8	-1.026033000	-0.751003000	3.950938000
1	-0.201021000	-0.361718000	3.594133000
16	-1.020796000	-3.890934000	1.616900000
8	0.374332000	-4.031709000	0.879310000
6	-1.116038000	-4.032816000	3.427937000
6	-2.532418000	-3.630761000	0.640700000
1	-0.789102000	-3.109663000	3.898981000

1	-2.581911000	-2.600899000	0.296958000
1	-0.523180000	-4.899716000	3.713210000
1	-2.170307000	-4.217085000	3.635977000
1	-3.344290000	-3.847134000	1.335700000
1	-2.525732000	-4.361439000	-0.165515000

TS reaction 10 (-2457.78256611):

12	-4.599569000	1.495932000	-0.232219000
8	-6.625530000	0.940660000	-0.641420000
8	-4.339264000	2.326930000	-2.188252000
8	-2.686737000	2.064939000	-0.114607000
8	-5.271997000	3.393474000	0.397424000
8	-4.035660000	-0.463837000	-0.879895000
1	-4.734715000	3.197461000	-2.310203000
1	-3.385409000	2.479689000	-2.071752000
16	-1.593432000	1.079730000	0.327629000
1	-6.155582000	3.422825000	0.779670000
1	-4.686695000	3.823249000	1.067284000
1	-4.031444000	-0.974776000	-0.057185000
1	-3.083621000	-0.452457000	-1.132666000
1	-6.857331000	0.438518000	0.153818000
1	-6.752828000	0.350005000	-1.392891000
8	-0.448047000	1.807853000	0.868146000
8	-1.332029000	0.122590000	-0.776816000
8	-2.278700000	0.171655000	1.431850000
16	-4.507137000	0.680250000	2.918967000
8	-5.206433000	1.630819000	3.790050000
8	-2.973785000	1.489088000	2.603480000
8	-4.190764000	-0.626343000	3.448489000

8	-5.117914000	0.644664000	1.539723000
16	-3.105350000	3.457034000	3.338665000
6	-3.504060000	3.867646000	5.058658000
1	-2.943876000	3.186058000	5.698199000
1	-3.235752000	4.910105000	5.234610000
1	-4.571862000	3.690743000	5.160294000
6	-1.336778000	3.738204000	3.211506000
1	-1.155473000	4.809473000	3.297513000
1	-0.854297000	3.175151000	4.010653000
1	-1.020483000	3.334959000	2.245213000
8	-3.829270000	4.427194000	2.439758000

TS reaction 12 (-2212.05723822):

12	-4.779771000	1.055711000	-1.094142000
8	-6.259203000	-0.086513000	-2.127684000
8	-4.662740000	2.410606000	-2.734031000
8	-3.577478000	2.472949000	-0.237136000
8	-6.343138000	2.059136000	-0.066947000
8	-3.128814000	0.024994000	-1.930925000
1	-4.324865000	2.062483000	-3.566952000
1	-4.015143000	3.056246000	-2.414721000
16	-2.157290000	2.206846000	0.229113000
1	-6.672868000	1.452080000	0.638408000
1	-5.900224000	2.772356000	0.411650000
1	-3.053715000	-0.786115000	-1.410419000
1	-2.308298000	0.540078000	-1.734393000
1	-6.349625000	-0.808049000	-1.484657000
1	-7.137441000	0.286603000	-2.260078000
8	-1.622762000	3.314347000	1.026208000

8	-1.338114000	1.797593000	-0.947467000
8	-2.278034000	0.919529000	1.140193000
16	-5.166663000	-0.413672000	1.631282000
8	-6.589507000	0.067314000	1.803290000
8	-4.376097000	0.827560000	2.088527000
8	-4.826743000	-1.628101000	2.353837000
8	-4.907172000	-0.554842000	0.130745000
6	-0.033060000	3.779591000	4.963284000
6	-1.359847000	4.237405000	5.137757000
6	0.217383000	2.670273000	4.199707000
1	-1.540689000	5.120014000	5.739652000
1	1.216928000	2.292362000	4.029407000
6	-2.437534000	3.567917000	4.547013000
6	-0.874137000	1.981133000	3.583220000
1	-3.446594000	3.930243000	4.696772000
6	-2.221334000	2.451468000	3.770304000
1	-3.033574000	1.903537000	3.309676000
1	0.782854000	4.316370000	5.431023000
8	-0.620543000	0.921774000	2.885789000
1	-1.390474000	0.773269000	2.155809000
